# Effect of Medical Chitosan on Clinical Efficacy and Pain in Knee Osteoarthritis: A Systematic Review and Meta-Analysis

**DOI:** 10.3390/diseases14070252

**Published:** 2026-07-14

**Authors:** Qiao Lian, Li-Bing Liang, Cai-Qin Wu, Yan Zhu, Kun-Peng Li

**Affiliations:** 1 School of Exercise and Health, Shanghai University of Sport, Shanghai 200438, China; 23600516@sus.edu.cn; 2 School of Nursing, Shanghai University of Traditional Chinese Medicine, Shanghai 201203, China; 22024483@shutcm.edu.cn (L.-B.L.); wucaiqin@shutcm.edu.cn (C.-Q.W.); 3 Department of Neurological Rehabilitation, Shanghai Second Rehabilitation Hospital, Shanghai 200431, China

**Keywords:** chitosan, knee osteoarthritis, pain relief, clinical efficacy, systematic review

## Abstract

Background: Knee osteoarthritis (KOA) affects over 300 million people globally, yet current therapies face limitations in efficacy and safety. Chitosan, a natural polysaccharide, has shown conflicting evidence in KOA management. This systematic review evaluates chitosan’s clinical effectiveness and defines its optimal application scenarios. Methods: Randomized controlled trials (RCTs) published before the end of November 2024 on chitosan and knee osteoarthritis were systematically retrieved from PubMed, Web of Science, Cochrane Library, EBSCO, PsycINFO, ClinicalTrials.gov, SINOMED, Chinese Medical Journal Network, CNKI, VIP, Wanfang Data, Embase, and SCOPUS. After screening the references, publications meeting the inclusion and exclusion criteria was selected. RevMan5.4 software was used to perform the meta-analysis of the data. Results: A total of 231 articles were retrieved, and 14 RCTs involving 1504 participants were included. For the primary efficacy outcome, pooled analysis of 10 RCTs showed a significantly higher overall clinical response rate in the chitosan group compared with the control group (OR = 5.43, 95% CI = 3.21, 9.18, *p* < 0.001), with no observed heterogeneity (*I*^2^ = 0%). Additionally, meta-analysis of 12 RCTs demonstrated a significant reduction in visual analog scale (VAS) pain scores (MD = −1.06, 95% CI = −1.38, −0.73, *p* < 0.001) in patients receiving intra-articular chitosan injection. Subgroup analysis of VAS scores showed that pain reduction varied significantly by follow-up duration (*I*^2^ for subgroup differences = 62.5%, *p* = 0.03). Conclusions: Intra-articular injection of medical chitosan shows potential benefits for pain relief and clinical efficacy in patients with knee osteoarthritis, with a low incidence of adverse reactions. Nevertheless, the evidence remains promising but preliminary.

## 1. Introduction

Knee osteoarthritis (KOA) is a common degenerative joint condition with significant global health impacts. In 2019, there were roughly 364.6 million global KOA cases, and the age-adjusted prevalence rate reached 4376 cases per 100,000 people [1]. For adults aged 65 and above, KOA is one of the main causes of disabilities [2]. KOA prevalence varies sharply by region, with the Western Pacific region having a notably high rate of 7319.87 cases per 100,000 population. Gender differences are also obvious, as women are more likely to develop KOA than men in most countries. The disease becomes more common with increasing age [3]. In 2019 alone, there were an estimated 29.5 million new KOA cases, with an age-standardized incidence rate of 350.3 per 100,000 people, which shows the disease’s growing global burden [4].

The key challenge in KOA management is the irreversible progressive destruction of articular cartilage, which eventually causes pain and functional impairment [5]. Currently, three main treatment methods are used in clinical practice: non-pharmacological therapies, pharmacological therapies, and surgery for severe cases. Non-pharmacological treatments, like exercise therapy [6] and physical therapy [7], can improve joint function to some extent. But KOA is essentially a chronic degenerative joint disease [8], so non-pharmacological therapies are less effective in the later stages. As the disease progresses, joint damage continues to worsen, making drug therapy a necessary intervention at this stage. Due to its invasiveness, higher cost, and potential complications, surgical intervention such as total knee arthroplasty is generally reserved for patients with end-stage KOA who have failed conservative treatments [5,9]. Therefore, pharmacological therapies are prioritized in the stepwise management of KOA, especially for mild-to-moderate cases, as they can effectively control symptoms and delay disease progression while minimizing surgical risks [5,10,11,12].

The main pharmacological treatment options for KOA include nonsteroidal anti-inflammatory drugs (NSAIDs), intra-articular corticosteroid injections, hyaluronic acid, and medical chitosan.

Chitin, a natural biopolymer polysaccharide, has shown potential therapeutic value in relevant studies [13]. Chitosan-based fiber mats have been shown to support chondrocyte adhesion and proliferation, a characteristic that further aids in their role of protecting cartilage [14]. Intra-articular injection of carboxymethyl chitosan has been confirmed to provide rapid pain relief within 1 week and sustained efficacy for up to 9 months, with additional improvements in knee function observed [15]. For patients with advanced knee osteoarthritis who show no response to hyaluronic acid treatment, intra-articular injection of carboxymethyl chitosan significantly improves pain and joint function-related symptoms such as stiffness, crepitus during movement, and swelling. This intervention not only addresses the unmet clinical need in patients unresponsive to hyaluronic acid therapy but also demonstrates its value as a complementary treatment [16].

That said, the use of chitosan still sparks controversy. For instance, a 12-month study on carboxymethyl chitosan found that the treatment works better for patients with early or mild KOA, whereas its effectiveness lessens in those with severe disease [17]. What is more, the quality of chitosan-based treatments depends on three key factors: the source of chitosan, its molecular weight, and the delivery method used [14]. Looking at recent research, a relevant review points out that it is still unclear whether carboxymethyl chitosan offers long-term advantages over a placebo. Even so, a small trial focusing on KiOmedine^®^ CM-Chitosan did report notable short-term improvements [18,19].

When it comes to exploring chitosan, particularly for KOA, clinicians and researchers still have wide-ranging debates over whether this substance ought to be integrated into standard treatment protocols in clinical practice. What clinical data reveals is that carboxymethyl chitosan serves as a minimally invasive option, and it brings safety advantages for patients who either cannot undergo other treatments or find they are unable to tolerate them [15]. Relevant reviews, though, point out that evidence to back up the regular use of carboxymethyl chitosan is still insufficient [19]. Adding to this challenge, differences in chitosan’s source materials and manufacturing processes, such as variations in deacetylation level, viscosity, and endotoxin amounts, have been shown to trigger inconsistent biological reactions [20]. This lack of standardization does not just make it more difficult to interpret research on carboxymethyl chitosan; it also slows down the process of reaching clear conclusions about how effective it is. If we aim to figure out what role carboxymethyl chitosan should play in KOA treatment, pulling together the mixed evidence available right now is viewed as a necessary step. This approach follows the same systematic method that was applied earlier when researchers studied glucosamine and chondroitin [21].

To address these gaps, a systematic review and meta-analysis has been conducted following PRISMA guideline principles [22], with the literature on chitosan in KOA collated and synthesized. By synthesizing existing data, this review aimed to elucidate the role of chitosan in the treatment of knee osteoarthritis, thereby providing evidence-based guidance for clinicians. The present study is highly consistent with the findings of the Global Burden of Disease Study, which explicitly indicates that the global prevalence of arthritis has exhibited a sustained upward trend [23], and the economic burden induced by this condition has become increasingly prominent alongside the expansion of its epidemiological scope [24]. Collectively, this research underscores the urgency and necessity of implementing such evidence-based intervention strategies.

## 2. Methods

### 2.1. Protocol and Guidance

This systematic review and meta-analysis is reported according to the Preferred Reporting Items for Systematic Reviews and Meta-Analyses (PRISMA) reporting guideline. This systematic review was prospectively registered in PROSPERO (CRD420251163609).

### 2.2. Databases and Search Strategies

A search of the literature was conducted in PubMed, Web of Science, Cochrane Library, EBSCO, PsycINFO, ClinicalTrials.gov, SINOMED, Chinese Medical Journal Network, CNKI, VIP, Wanfang Data, Embase, and Scopus, covering the period from each database’s inception to 5 November 2024. Search terms used were knee osteoarthritis and chitosan. We included relevant synonyms to expand our search for titles, abstracts, and keywords in those databases. Eligible articles were identified through keyword and entry term searches, along with manual searching. Search words included (“knee osteoarthritis”, “Knee Osteoarthritides”, “KOA”, “Knee Osteoarthritis”, “Osteoarthritis of Knee”, “Osteoarthritis of the Knee”, “Osteoarthritis of the knee joint”, “osteoarthritis of articular genu”) AND (“chitosan”, “Poliglusam”, “Medical-grade Chitosan”, “Medical Chitin-derivative”, “Medical Chitosan”, “Chitosan for Medical Use”, “Medical hexose”, “Medical chitose”). The full search strategies for all databases are provided in Supplementary File S1.

### 2.3. Study Selection

The inclusion criteria were as follows. (1) Study type: Randomized controlled trial (RCT). (2) Study subjects: Patients meeting medical diagnostic criteria for knee osteoarthritis (knee osteoarthritis, knee osteoarthrosis) and experiencing pain. The diagnostic basis includes the “Guidelines for Diagnosis and Treatment of Osteoarthritis” of the Orthopedic Society of China, the standards of the American College of Rheumatology (ACR), or the Kellgren–Lawrence (K-L) radiological grading criteria (grades I–IV). These are considered to be authoritative standards. (3) Intervention measures: The intervention group received single treatment of intra-articular injection of medical chitosan or treatment combined with other treatments; the control group received conventional treatment without medical chitosan.

The exclusion criteria were as follows. (1) Duplicate publications: We removed duplicate publications or articles with duplicated data through EndNote. (2) Missing data: Publications where the full text or key outcome indicators cannot be obtained or those where the original data are incomplete. (3) Inconsistent content: Research topics not related to “medical chitosan treatment for knee osteoarthritis” or where the intervention/control measures are not clear. Relevant articles retrieved from these databases were exported to bibliographies and then imported into EndNote to eliminate duplication. Articles were initially screened by their titles and abstracts for inclusion, with further evaluation done by reading the full text. Those not meeting the criteria were excluded.

### 2.4. Outcome Indicator

The primary outcomes were pain intensity assessed by the visual analog scale (VAS) and clinical efficacy (total effective rate). Secondary outcomes included joint function, range of motion (ROM), safety, and disease progression (K-L grading). This classification follows the OMERACT-OARSI core domain set for knee osteoarthritis [25].

The main outcome measures are outlined as follows. ① Primary outcome: Pain intensity is primarily assessed using the VAS. To provide comprehensive reference data, supplementary assessment tools are also adopted for pain evaluation, including the Numerical Rating Scale (NRS), McGill Pain Questionnaire (MPQ), and Lequesne Index. ② Primary outcome: Clinical efficacy is measured using the total effective rate as the statistical metric. ③ Joint function assessment: This assessment incorporates multiple scales, such as the Western Ontario and McMaster Universities Osteoarthritis Index (WOMAC), the Lequesne Index, Lysholm score, Rasmussen score, and American Knee Society score (AKS). ④ Safety indicators: The incidence of adverse reactions is analyzed statistically, with monitored reactions including joint swelling and pain reactions. ⑤ Range of motion (ROM): The flexion and extension angles of the knee joint are measured. ⑥ Disease progression-related indicators: The Kellgren–Lawrence (K-L) grading system is used to assess the degree of joint degeneration.

### 2.5. Classification of Control Interventions

The control interventions in the 14 included RCTs were categorized into seven types: (1) conventional physical therapy combined with oral NSAIDs; (2) intra-articular corticosteroids; (3) oral or topical NSAIDs alone; (4) intra-articular hyaluronic acid with or without oral NSAIDs; (5) ozone irrigation alone; (6) traditional Chinese medicine external wash; and (7) no pharmacological intervention (health education only).

### 2.6. Methodological Quality Assessment

Two researchers independently assessed the methodological quality of the included RCTs using the Cochrane risk-of-bias 2.0 (RoB 2.0) tool. For each domain, the risk of bias was judged as low risk, high risk, or some concerns based on the RoB 2.0 signaling questions. Because none of the included studies reported allocation concealment or blinding of participants and personnel, these domains were rated as high risk of bias. Other potential biases (funding, co-interventions, center effects) were examined, and no major concerns were found. Inter-rater reliability for the randomization process domain was assessed using Cohen ‘s kappa (κ = 0.86, 95% CI 0.66–1.00), indicating almost perfect agreement. The raw assessment data are provided in Supplementary Table S1.

### 2.7. Data Extraction

Two researchers conducted screening of the literature and data extraction independently, cross-verifying their findings. When disagreements arose, a third researcher helped achieve consensus. The extracted data encompassed information such as the primary author, publication year, study design, number of original studies incorporated, participant count, interventions, outcome metrics, and tools for assessing methodological quality [26].

### 2.8. Statistical Analyses

RevMan 5.4 was used to perform all meta-analysis. Despite methodological limitations in some included studies, quantitative meta-analysis was performed to provide pooled effect estimates with greater statistical power and precision while acknowledging heterogeneity and conducting sensitivity analyses to test robustness. For continuous outcomes, including VAS, WOMAC, and Lequesne, the mean difference (MD) was used as the effect measure. For the dichotomous outcome of clinical efficacy, the odds ratio (OR) was used. The choice of meta-analytic model was based on the *I*^2^ statistic: a random effects model was used when *I*^2^ exceeded 50% (indicating substantial heterogeneity), and a fixed effect model was applied when heterogeneity was low (*I*^2^ ≤ 50%). Subgroup analyses by follow-up duration were performed for VAS, Lequesne, and WOMAC to explore potential sources of heterogeneity. Sensitivity analysis was conducted using a leave-one-out approach for the primary outcomes (VAS and clinical efficacy) to test the robustness of the pooled estimates. The certainty of evidence for each outcome was assessed using the GRADE framework, with results presented in Supplementary Tables S1 and S2. A two-tailed *p*-value (*p* < 0.05) was considered statistically significant.

## 3. Results

### 3.1. General Characteristics of Included Studies

**Figure 1:**
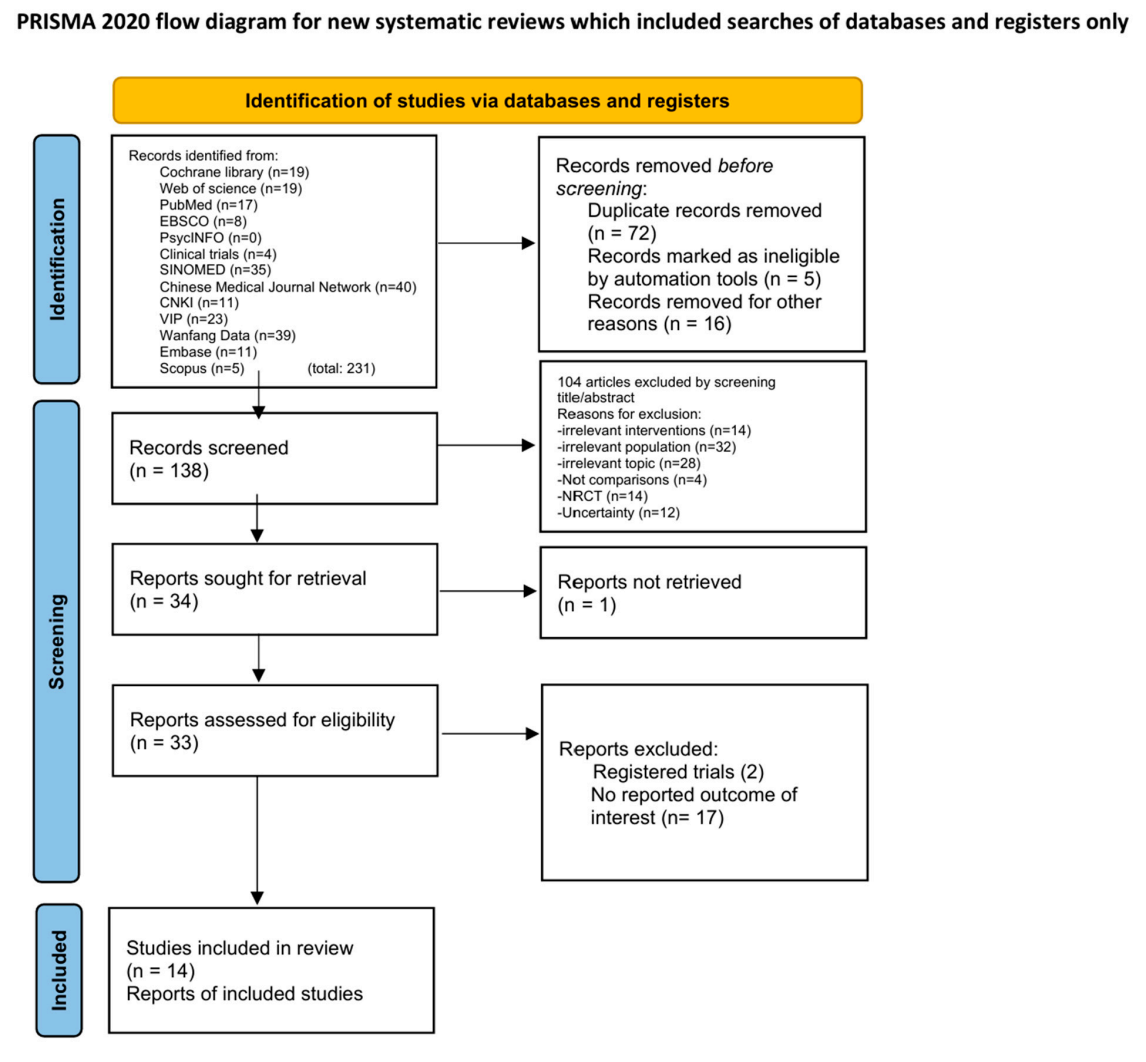
PRISMA flow diagram.

The study selection process was summarized in the PRISMA flowchart (Figure 1). A total of 231 relevant articles were initially identified, with 148 in Chinese and 83 in English. Next, 93 publications were excluded; these included duplicates, cross-referenced articles, and those that clearly failed to meet the inclusion criteria. After this step, 33 clinical control studies were selected by reviewing titles and abstracts. Further steps involved searching full texts, conducting full-text reviews, and performing quality evaluations. Through these processes, 19 studies were excluded; the excluded studies comprised non-randomized controlled trials, clinical trials without control groups, and studies where the authenticity of being randomized controlled trials (RCTs) could not be confirmed. In total, 14 RCTs were included, with 13 in Chinese and one in English. The basic characteristics of the included RCTs are shown in Table 1.

Regarding the reporting of outcome indicators, 12 of the 14 included RCTs reported VAS pain scores [27,29,30,32,33,34,35,36,37,38,39,40]. Six RCTs [30,31,32,33,35,38] reported WOMAC scores. One RCT [39] reported joint range of motion. Five RCTs [27,31,34,37,38] reported the Lequesne index. A total of 10 RCTs [27,28,29,32,34,35,36,37,38,39] reported clinical efficacy. Four RCTs [33,36,38,40] reported safety indicators.

### 3.2. Methodological Quality Assessment

The quality of the RCTs was evaluated and graded according to the quality assessment criteria in the *Cochrane Reviewer’s Handbook* [41]. Among the 14 included RCTs, only one [33] adopted a double-blind design, while the rest mostly only achieved blinding of the evaluators. Regarding the randomization method, eight were clearly described, and six were not elaborated upon; all studies had no selective reporting bias. Overall, there were two high-quality RCTs, six of moderate quality, and six of low quality.

### 3.3. Primary Outcome

#### 3.3.1. Pain

The Visual Analog Scale

**Figure 2:**
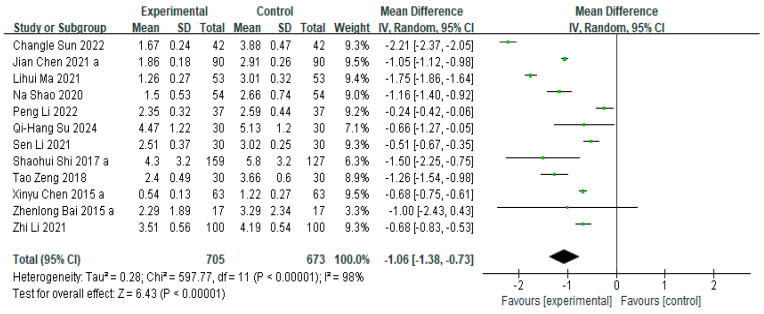
Forest plot of overall VAS scores after intra-articular chitosan injection versus control [27,28,29,31,32,34,35,36,37,38,39,40].

A meta-analysis of 12 RCTs (1378 participants) demonstrated that chitosan significantly reduced VAS scores compared with controls (MD = −1.06, 95% CI = −1.38, −0.73, *p* < 0.001, *I*^2^ = 98%) (Figure 2).

**Figure 3:**
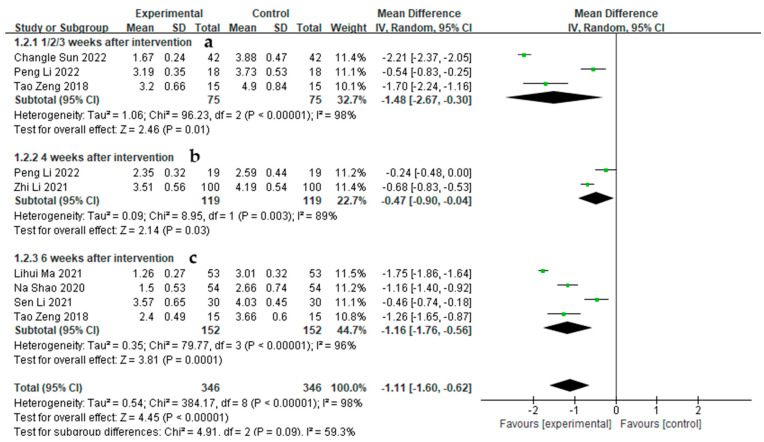
Subgroup analysis of VAS scores by follow-up duration: (**a**) 1–3 weeks after intervention; (**b**) 4 weeks after intervention; (**c**) 6 weeks after intervention [27,28,31,32,35,37,38].

**Figure 4:**
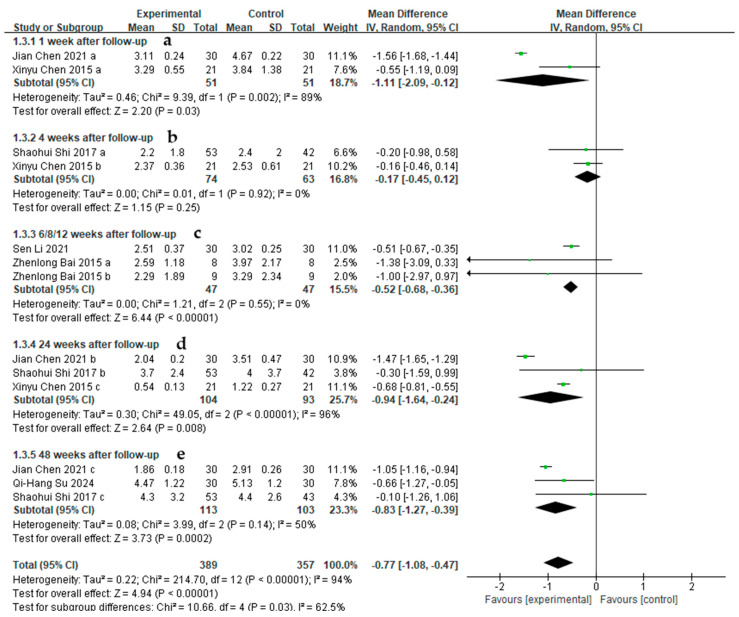
Subgroup analysis of VAS scores by follow-up duration: (**a**) 1 week; (**b**) 4 weeks; (**c**) 6–12 weeks; (**d**) 24 weeks; (**e**) 48 weeks [29,31,34,36,39,40].

For immediate post-intervention results, analysis of seven RCTs (692 participants) showed lower VAS scores in the chitosan group (MD = −1.11, 95%CI = −1.60, −0.62, *p* < 0.001, *I*^2^ = 98%) (Figure 3). For follow-up results, analysis of six RCTs (746 participants) also demonstrated consistently lower VAS scores in the chitosan group (MD = −0.77, 95%CI = −1.08, −0.47, *p* < 0.001, *I*^2^ = 94%) (Figure 4).

Because some studies reported both immediate and follow-up VAS scores, the participant counts in the subgroup analyses are not mutually exclusive and sum to more than the overall total.

This RCT [31] demonstrated that the intervention group showed notably greater improvements in pain than the control group after 4 weeks of follow-up (*p* < 0.050).

In one RCT [28], 1 week, 3 weeks, and 5 weeks post-follow-up, the intervention group’s NRS scores were all significantly lower than the control group’s scores at the corresponding time points (*p* < 0.050).

In one RCT [38], the MPQ score of the intervention group after treatment was significantly lower than that of the control group (*p* < 0.050).

#### 3.3.2. Evaluation of Clinical Efficacy

**Figure 5:**
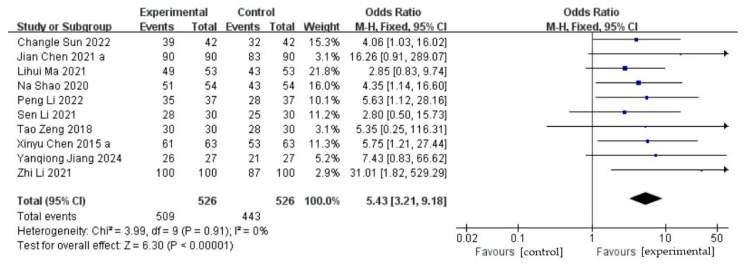
Forest plot of clinical efficacy [27,28,29,30,31,32,34,35,37,38].

Overall, across all 10 RCTs, the odds ratio of the intervention group was higher than that of the control group (OR = 5.43, 95%CI = 3.21, 9.18, *p* < 0.001, *I*^2^ = 0%) (Figure 5).

### 3.4. Secondary Outcome

#### 3.4.1. Lysholm Score

In Shi et al.’s RCT [40], compared with before treatment, the Lysholm score of the patients in the intervention group significantly increased after 1, 6, and 12 months of treatment (F = 8.749, *p* < 0.050).

#### 3.4.2. Knee Joint Function Score

Two RCTs [28,29] demonstrated that the knee joint function scores of the intervention group were significantly lower than those of the control group at the same period (*p* < 0.050). In Li et al.’s RCT [36], the Rasmussen score of the intervention group was significantly higher than that of the control group (*p* < 0.001).

#### 3.4.3. Lequesne Index Score

**Figure 6:**
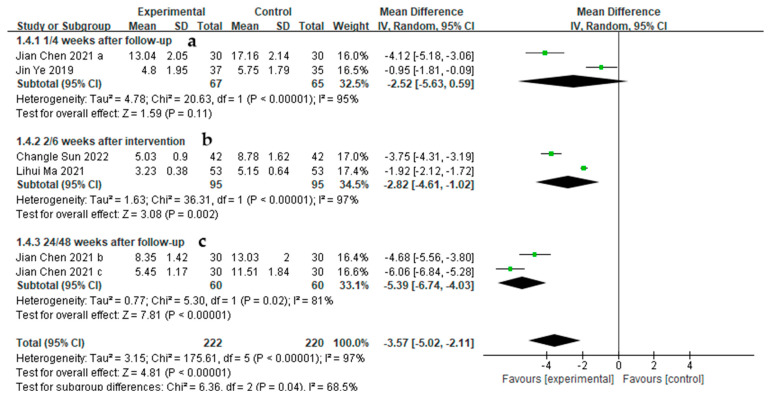
Subgroup analysis of Lequesne index by follow-up duration: (**a**) 1–4 weeks after follow-up; (**b**) 2–6 weeks after intervention; (**c**) 24–48 weeks after follow-up [29,32,33,38].

Subgroup analysis of Lequesne index by follow-up duration (Figure 6) showed that chitosan significantly reduced Lequesne scores overall compared with controls (MD = −3.57, 95%CI = −5.02, −2.11, *p* < 0.001, *I*^2^ = 97%) (Figure 6).

#### 3.4.4. WOMAC

**Figure 7:**
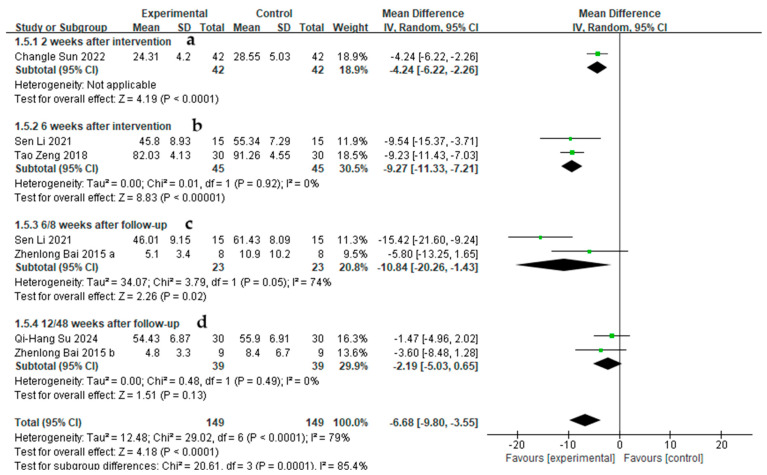
Subgroup analysis of WOMAC score by follow-up duration: (**a**) 2 weeks after intervention; (**b**) 6 weeks after intervention; (**c**) 6/8 weeks after follow-up; (**d**) 12/48 weeks after follow-up [31,32,37,39,40].

Subgroup analysis of WOMAC score by follow-up duration (Figure 7) showed that chitosan significantly reduced WOMAC scores overall compared with controls (MD = −6.68, 95%CI = −9.80, −3.55, *p* < 0.001, *I*^2^ = 79%) (Figure 7).

#### 3.4.5. AKS

In this RCT [30], the AKS knee joint sub-score in the medical chitosan intervention group (CSI group) decreased significantly compared to the control group (OA group) (*p* < 0.050), but no significant difference was observed in the AKS functional sub-score between the two groups.

#### 3.4.6. Range of Motion

In the RCT [39], after completing the treatment course of receiving intra-articular injection of medical chitosan once every 2 weeks, three consecutive times, the knee joint range of motion of the intervention group was significantly higher than that of the control group (*p* < 0.050).

#### 3.4.7. Impact on Quality of Life

In this RCT [28], after follow-up, the observation group had significantly higher quality-of-life scores in the dimensions of physical health, psychological function, physiological function, social relationships, and emotional activities compared to the control group (*p* < 0.050).

#### 3.4.8. Kellgren–Lawrence Grading Results

In this RCT [30], there was no significant difference in the postoperative K-L grades between the two groups (*p* > 0.050).

#### 3.4.9. Occurrence of Adverse Reactions

The adverse reactions included nausea, abdominal distension, and other symptoms. In this RCT [36], the adverse reaction rate in the intervention group was significantly lower than that in the control group (*p* = 0.009). In one RCT [33], the adverse reaction rate of the intervention group showed no significant difference compared with the control group (*p* > 0.050); all adverse reactions did not affect the treatment process.

#### 3.4.10. Complications of Joint Aspiration

One RCT [38] showed there was no statistically significant difference between the two groups (*χ*^2^ = 0.155, *p* > 0.050). In the RCT [40], the total incidence in the medical chitosan group was lower than that in the sodium hyaluronate group (*p* < 0.050), when incidence was calculated by the number of punctures, with no statistically significant difference between them (*p* > 0.050). All complications in the intervention group were mild, with no serious complications observed.

### 3.5. Sensitivity Analysis

Leave-one-out sensitivity analyses showed that the pooled effect estimates remained statistically significant after excluding each individual study. For VAS, the mean difference ranged from −1.14 to −0.93 (all *p* < 0.001). For clinical efficacy, the odds ratio ranged from 4.66 to 6.14 (all *p* < 0.001). These results confirm that the conclusions are robust and not driven by any single trial (Supplementary Figures S1 and S2).

### 3.6. GRADE Assessment

The certainty of evidence was moderate for clinical efficacy and very low for VAS, Lequesne, and WOMAC scores. The downgrading factors included serious risk of bias, very serious inconsistency, and for the functional outcomes, serious imprecision (Supplementary Tables S1 and S2).

### 3.7. Publication Bias

Funnel plot analysis revealed marked asymmetry for the outcomes of VAS score and clinical efficacy, indicating the presence of significant publication bias. Detailed funnel plots are provided in Supplementary Figures S3 and S4.

## 4. Discussion

### 4.1. Summary of Findings

This study elucidates that intra-articular injection of medical chitosan exerts a significant mitigating effect on VAS scores in patients with knee osteoarthritis, outperforming the control group. It further yields notable improvements in WOMAC scores, Lequesne index, and knee range of motion, while markedly elevating the efficacy of clinical evaluation outcomes. Notably, only mild localized adverse reactions at the injection site were documented throughout the study. It also found that both groups had elevated postoperative K-L grades.

### 4.2. External Validity and Reasons for Limited International Adoption

Most included studies (13/14) were conducted in China and published in Chinese journals, which limits generalizability to other populations. Unsurprisingly, intra-articular chitosan has not yet gained widespread acceptance internationally, particularly in Western countries.

Several factors likely explain this gap. First, the evidence base is geographically confined, with no independent multicenter trials outside China. Second, most existing RCTs have small sample sizes and methodological limitations, such as lack of blinding and inadequate allocation concealment. Third, direct head-to-head comparisons with established injectables, such as hyaluronic acid (HA) and platelet-rich plasma (PRP), are lacking. HA and PRP have been rigorously tested in large-scale, high-quality studies and are recommended in major guidelines [5,42]. Fourth, regulatory and market factors may have favored existing viscosupplements.

Therefore, while chitosan appears promising, its current role should be considered adjunctive rather than first-line, especially where better-established alternatives are readily available. Future research should prioritize high-quality, multicenter trials with rigorous methodology, especially in Western populations, and direct comparisons with standard injectables.

### 4.3. Effects of Medical Chitosan on Pain

To systematically clarify the efficacy of medical chitosan (alone or in combination with other interventions) in alleviating pain in patients with KOA, this analysis centers on three core pain assessment tools: VAS, NRS, and MPQ.

For the primary indicator of pain intensity, the VAS, both data sets demonstrated significantly lower VAS scores in the medical chitosan group, accompanied by high heterogeneity. This heterogeneity is primarily attributable to inter-study variations in injection frequency, dosage, treatment duration, and follow-up periods, factors that likely underpin the observed variability in the magnitude of VAS score reduction. In addition, dose and injection frequency were nearly uniform across studies (13/14 used 2 mL and multiple injections), and K-L grade was inconsistently reported, making subgroup analysis for these factors unfeasible.

For the NRS, one RCT [28] demonstrated that the >20% reduction in scores relative to the baseline aligns with the efficacy threshold of standard KOA pain interventions, such as intra-articular hyaluronic acid, underscoring the value of this combined intervention for short-term pain relief in this patient population.

For MPQ, one RCT [38] shows that medical chitosan combined with minimally invasive surgery significantly reduced postoperative scores. This reduction links to synovial fluid restoration and inflammation suppression, consistent with findings from chitosan hydrogel studies [14].

Mechanistically, VAS, NRS, and MPQ reductions all relate to medical chitosan’s synergistic anti-inflammatory, lubricating, and neuron-modulating effects. Its suppression of IL-1β and TNF-α [43] blocks inflammation-mediated pain pathways [44,45], activates dorsal root ganglion neurons [44,46], and upregulates TRPV1 [47] to amplify pain signals. Medical chitosan’s viscoelasticity also forms a joint lubricating barrier [48], reducing cartilage friction and mechanical stress to further lower VAS and NRS scores [49]. For combined interventions, exercise enhances quadriceps strength to complement lubrication [28], and ozone injection modulates NF-κB [38,50] to reinforce anti-inflammatory effects, specifically improving MPQ-assessed pain quality.

In practice, chitosan is only effective for short-term relief or, when combined with other interventions, for sustained benefits, particularly in complex cases such as mid-stage or senile KOA. Optimization directions include standardizing intervention protocols and follow-up schedules to reduce VAS heterogeneity, conducting 3–6-month longitudinal RCTs to validate sustained improvements in NRS and MPQ outcomes, and designing factorial trials to quantify the independent role of medical chitosan in multimodal MPQ interventions [14,28,38,43,44,45,46,47,48].

### 4.4. Effects of Medical Chitosan on Knee Function in Patients with Knee Osteoarthritis

This study first establishes six key functional indicators for evaluation: Lysholm score, knee function score, Lequesne index score, WOMAC score, AKS score, and ROM.

For collective effect positioning, all six key indicators confirm medical chitosan’s consistent efficacy in enhancing KOA patients’ knee function: the Lysholm score improves post-treatment to levels comparable to arthroscopic intervention [40,51], and the knee joint function score is significantly better than that of controls [27,32,34].

Notably, pain and activity limitation stand as key factors in daily functional capacity, and both saw clear improvement. The Lequesne Index recorded significantly lower scores in the medical chitosan-only group [31,34,37,38]. In the meantime, the medical chitosan group also showed meaningful gains in the WOMAC, a tool crafted to evaluate pain, stiffness, and physical function.

Such high *I*^2^ values indicate substantial heterogeneity, which stems from variations in intervention duration, follow-up time, and treatment courses, where intervention duration varies with diverse medical chitosan injection schedules, follow-up time ranges from weeks to months, and treatment courses include combinations with other therapies or different oral medication durations.

Carboxymethyl chitosan, a derivative of medical chitosan, leads to further reductions in WOMAC scores at the 3-month time point [30,32,33,35,38]. Additionally, the AKS joint sub-score exhibited a significant decrease [30]. Meanwhile, the medical chitosan group, which received specific treatment, showed a higher ROM than the control group receiving conventional therapy [39,52,53]. These findings collectively demonstrate that carboxymethyl chitosan, a chitosan derivative, exerts multidimensional therapeutic effects in patients with knee osteoarthritis (KOA).

In terms of mechanism, by inhibiting the NF-κB pathway, medical chitosan blocks the secretion of IL-1β and TNF-α in human chondrocytes [43], while chitosan oligosaccharides activate AMPK, reducing TNF-α-induced iNOS/COX-2 expression in synovial cells [54]. These effects alleviate synovial inflammation, thereby reducing the Lequesne index and the WOMAC. Medical chitosan promotes chondrocyte adhesion and proliferation [55], thereby maintaining the structural integrity of the joints. This protective action further enhances the Lysholm scores and AKS joint sub-scores.

In enhancing joint lubrication, chitosan forms a lubricating film with lower friction than hyaluronic acid, and intra-articular injection restores synovial fluid viscoelasticity [56]. Additionally, combining medical chitosan with exercise boosts muscle strength and joint coordination, further improving functional indicators like the knee joint function score [32,34,55].

Practically, the efficacy of medical chitosan across indicators makes it a valuable non-surgical option for KOA patients, especially elderly individuals with comorbidities, given its equivalence to arthroscopic intervention for the Lysholm score. Optimization directions include conducting longer RCTs of 2–3 years to confirm sustained effects, standardizing scoring criteria and intervention protocols for the knee joint function score, reporting WOMAC sub-score data, and exploring combination strategies, such as medical chitosan plus targeted exercise, to address gaps, such as the AKS functional sub-score [17,30,34,57,58].

### 4.5. Evaluation of Clinical Efficacy

For knee osteoarthritis, two of 12 RCTs failed to provide precise rates of clinical efficacy. A meta-analysis of the remaining 10 RCTs demonstrated that medical chitosan exhibits significant therapeutic advantages over control groups (*p* < 0.001, *I*^2^ = 0%) (Figure 5). Its efficacy comes from medical chitosan’s ability to block the activity of proinflammatory cytokines in chondrocytes and synovial tissue, which, in turn, helps lower joint damage [43].

This efficacy is consistent with findings from other chitosan-based research, demonstrating that intra-articular injection of chitosan hydrogel enhances the viscoelasticity of synovial fluid, improves joint lubrication, and reduces friction between joint structures, thereby inhibiting inflammation and ultimately achieving analgesic effects with superior therapeutic efficacy compared to the control group [14]. Another study showed that chitosan achieved a higher overall efficacy rate than the control group, with sustained pain relief and improved joint function persisting for 6 months post-treatment [15]. Collectively, these findings corroborate the exceptional therapeutic efficacy of medical chitosan.

### 4.6. Impact on Quality of Life

An RCT found that the intervention group demonstrated significantly higher multidimensional quality-of-life scores [28]. This effect stemmed from the synergistic action of chitosan and exercise, which collectively improved quality of life [6]. This finding aligns with a study which demonstrated improvements in both physical functioning and emotional well-being after six months of treatment [15]. Furthermore, a systematic review on viscoelastic supplements combined with exercise therapy further supports this conclusion: effective interventions for knee osteoarthritis not only alleviate pain and enhance function but also significantly improve quality of life [5].

### 4.7. Kellgren–Lawrence Grading Results

In one RCT [30], both postoperative groups showed significant increases in K-L grading, indicating disease progression, with no intergroup differences, consistent with previous studies [17]. Another study on intra-articular medical chitosan also found ongoing structural degeneration, assessed by radiological grading, in both groups with no inter-group variation [15].

Mechanistically, K-L grading focuses on irreversible structural changes, such as joint space narrowing, osteophyte formation, and subchondral sclerosis [4], while medical chitosan primarily acts by inhibiting proinflammatory cytokines, enhancing synovial fluid viscoelasticity, and promoting chondrocyte adhesion [14,43]. These actions alleviate symptoms and delay further cartilage damage but are insufficient to reverse existing structural degeneration or halt the progression that elevates the K-L grades [9]. This aligns with findings that most non-surgical treatments, including Visco supplements, have a limited ability to slow radiological grade progression [5].

### 4.8. Safety Monitoring

#### 4.8.1. Occurrence of Adverse Reactions

One RCT [36] reported low-to-mild rates of adverse reactions associated with medical-grade chitosan. This finding lines up with a multicenter investigation that tracked outcomes of intra-articular carboxymethyl chitosan injections, a type of medical chitosan derivative. The multicenter study noted an overall adverse event rate below 5%, with any issues limited to mild local discomfort that did not force treatment to be stopped [15]. The low adverse reaction rate of medical-grade chitosan may be related to its biocompatibility, as it exhibits only minimal cytotoxicity toward human chondrocytes and synoviocytes [14].

Another RCT [33] found no significant difference in adverse reactions between the two groups, with neither affecting treatment outcomes. This conclusion is supported by a systematic review [5], indicating that chitosan-based products possess considerable safety. A preclinical study [54] using a rabbit knee osteoarthritis model confirmed the low-risk profile of medical chitosan; chitosan oligosaccharides suppressed proinflammatory cytokines without inducing abnormal local immune activation, explaining its mild adverse reaction. This conclusion requires further validation, necessitating additional large-scale clinical trials for confirmation.

#### 4.8.2. Complications of Joint Aspiration

In the two RCTs [38,40], the minor complications identified in the medical chitosan group were congruent with the safety profile of chitosan-based biomaterials, with only mild postoperative joint soreness documented. This finding is in accordance with the outcomes of in vitro studies [14].

No severe infections occurred in these groups, potentially attributable to the antimicrobial activity of chitosan, which disrupts bacterial cell membranes, inhibits microbial growth, and reduces the risk of infection [59]. No allergic reactions were observed in the intervention group, a phenomenon explained by the well-documented low immunogenicity of chitin [60]. Owing to its biological properties, this natural polysaccharide rarely triggers adverse immune responses. Statistically significant differences in complication rates were not observed between the medical-grade chitosan and sodium hyaluronate groups based on puncture frequency, although the total incidence was lower in the medical chitosan group. This finding aligns with viscoelastic supplement studies [11], which indicate that chitosan-based products offer puncture-related safety that is comparable to that of common joint injectables while potentially carrying a lower overall complication risk.

### 4.9. Search of the Literature and Quality Assessment

This study strictly followed the methodological guidelines for systematic reviews. It comprehensively searched 13 databases in both Chinese and English, including the Cochrane Library, etc. An initial 231 articles were identified, and after screening, only 14 RCTs were included, which indicates a relatively small number of eligible studies, most of which were in Chinese. The entire search process fully complied with the transparent reporting requirements of the PRISMA 2020 statement [26].

**Figure 8:**
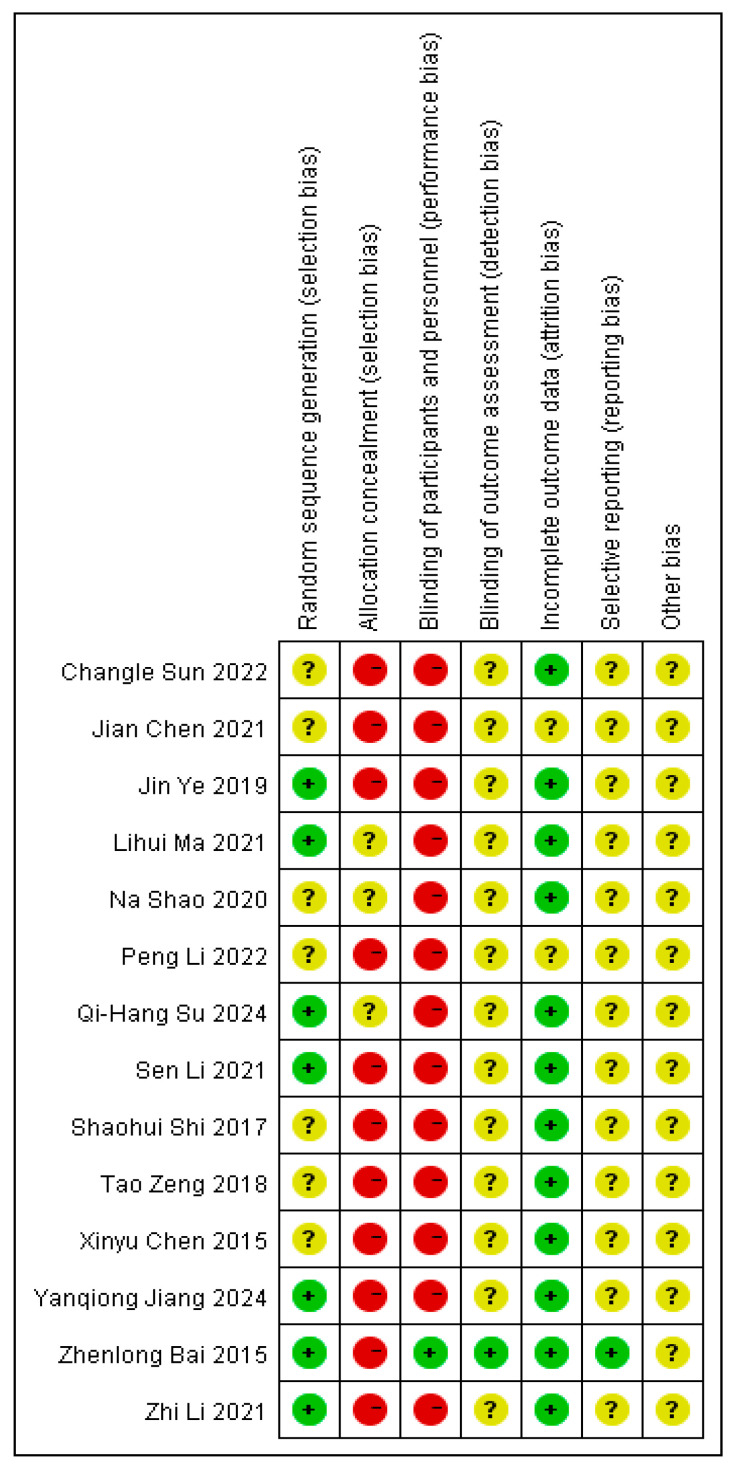
The risks of bias of included studies based on the Cochrane handbook [27,28,29,30,31,32,33,34,35,36,37,38,39,40].

The quality evaluation of the included RCTs using the Cochrane risk-of-bias assessment tool (ROB 2.0) revealed significant limitations in the methodology [41] (Figure 8). Only two studies clearly described the method for generating random sequences, and the studies did not report the allocation concealment scheme, which is a key design element to avoid selection bias; moreover, the studies did not implement blinding of outcome assessors, which might introduce detection bias. Additionally, seven studies had a sample size of less than 100 cases, which might reduce the statistical power and accuracy of the results [61].

The included RCTs had multiple methodological limitations: (1) only two studies [29,33] specified randomization methods, with no allocation concealment or outcome assessment blinding across all, failing to control bias [62]; (2) medical chitosan intervention protocols lacked standardization, showing fluctuations in dose, unadjusted combined therapy doses, varied injection frequency and treatment courses [63], hindering efficacy comparison; (3) some studies omitted WOMAC subscale data, and approximately 57% had a follow-up period of ≤6 months, with only four studies [30,34,38,40] including 12-month outcomes, which limits the evaluation of efficacy sustainability; (4) meta-analysis only synthesized VAS scores, while other indicators were only qualitatively described due to insufficient data, inconsistent criteria or divergent definitions; (5) the majority of studies had small sample sizes, with case numbers between 30 and 90, while only five studies [27,34,36,39,40] included 100 or more cases. Additionally, there were few patients classified as Kellgren–Lawrence grade IV, a factor that limits the ability to extrapolate results to severe cases [64].

### 4.10. Research Limitations and Prospects

#### 4.10.1. Limitations

This study has several limitations. First, the methodological quality of the included RCTs was generally low. Most studies lacked adequate randomization description, allocation concealment, and blinding, which may reduce the reliability of the conclusions. Second, the control interventions were highly heterogeneous, ranging from placebo-like comparators to active treatments such as NSAIDs, corticosteroids, and hyaluronic acid. In addition, substantial statistical heterogeneity was observed in most outcomes (*I*^2^ up to 98%), limiting the robustness of the pooled estimates. In addition, dose and injection frequency were nearly uniform across studies, making subgroup analysis for these factors unfeasible. Third, adverse event data were insufficient for quantitative meta-analysis because most studies had short or variable follow-up durations and did not systematically record adverse reactions; therefore, only a descriptive summary was provided. In addition, only VAS and clinical efficacy were quantitatively analyzed; other outcomes, such as Lequesne and WOMAC, were not pooled due to insufficient or inconsistently reported data, which may introduce outcome reporting bias. Fourth, many of the included studies were conducted in China and published in Chinese journals, which limits the generalizability of our findings to other populations and healthcare settings. These limitations reflect the quality of the original studies and highlight the need for more rigorous, large-scale, multicenter RCTs in the future.

#### 4.10.2. Prospects

Current evidence regarding medical chitosan for knee osteoarthritis remains preliminary. Although some included RCTs [30,37] suggest benefits in pain relief and function, the methodological quality of these trials is generally low. Future research should focus on the following aspects: (1) large-scale (*n* > 300), multicenter RCTs strictly adhering to CONSORT guidelines [61] for randomization, allocation concealment, and blinding; (2) standardizing chitosan formulation to enhance comparability, with a dose of 2 mL per session, administered biweekly for a total of three sessions; (3) employing core outcome measures, including VAS, WOMAC, and ROM, to evaluate long-term efficacy; (4) prioritizing chitosan monotherapy to minimize confounding from combined interventions; (5) conducting direct head-to-head comparisons with established injectables such as hyaluronic acid and PRP; (6) performing studies in diverse international populations, especially in Western settings. These priorities align with OARSI recommendations [5].

## 5. Conclusions

During the 1–12-month follow-up period, intra-articular injections of medical-grade chitosan were administered at a dose of 2 mL per session, every two weeks for a total of three sessions. This regimen was associated with reductions in pain, improvements in knee function and range of motion, and favorable overall clinical outcomes in patients with knee osteoarthritis, with a low incidence of complications. The current evidence is promising but still preliminary.

## Figures and Tables

**Table 1:** The basic characteristics of the included research.

Data Sources	Sample Size	Intervention Group	Control Group	Outcome Measures
Na Shao 2020 [27]	*n* = 108	54 cases: Intra-articular injection of medical chitosan (2 mL, once every 2 weeks, 3 times)	54 cases: Conventional physical therapy + oral non-steroidal anti-inflammatory drugs (NSAIDs)	VAS; ROM; total effective rate
Peng Li 2022 [28]	*n* = 74	37 cases: Intra-articular injection of medical chitosan (2 mL)	37 cases: Intra-articular injection of triamcinolone acetonide (40 mg per week)	VAS; knee function score; total effective rate
Jian Chen 2021 [29]	*n* = 180	90 cases: Intra-articular injection of medical chitosan (2 mL) + lidocaine (0.5 mL, 2%), once every 2 weeks, 2–3 times	90 cases: Oral diclofenac + topical Gutong plaster	VAS; Lequesne; clinical efficacy (excellent/good rate)
Yanqiong Jiang 2024 [30]	*n* = 54	27 cases: Intra-articular injection of medical chitosan (2 mL, 160 mg, once every 15 days, 2 times) + lidocaine + exercise prescription	27 cases: Conventional treatment (oral NSAIDs + physical therapy)	Total effective rate; NRS; knee function score; quality-of-life score
Sen Li 2021 [31]	*n* = 60	30 cases: Oral Kangjian Guanjiebao + intra-articular chitosan (2 mL, once every 2 weeks, 3 times) + quadriceps exercise	30 cases: Oral celecoxib + intra-articular sodium hyaluronate (2.5 mL, once a week, 5 times)	WOMAC; VAS; total effective rate
ChangLe Sun 2022 [32]	*n* = 84	42 cases: Acupotomy + intra-articular injection of O_3_ (30 μg/mL, 5 mL) + medical chitosan (2 mL), once every 2 weeks, 2 times	42 cases: Oral ibuprofen sustained-release capsules + glucosamine hydrochloride	VAS; MPQ; Lequesne; WOMAC; JOA
Jin Ye 2019 [33]	*n* = 72	37 cases: Intra-articular injection of medical chitosan (2 mL, once every 2 weeks) + oral diacerein (50 mg/day)	35 cases: Intra-articular injection of triamcinolone acetonide (40 mg/week) + oral glucosamine hydrochloride (80 mg, 3 times/day)	WOMAC; Lequesne
Xinyu Chen 2015 [34]	*n* = 126	63 cases: Medical chitosan alone	63 cases: Ozone irrigation alone	VAS; Lequesne; total effective rate
Zhi Li 2021 [35]	*n* = 200	100 cases: Intra-articular injection of compound betamethasone (1 mL, once every 2 weeks) + medical chitosan (2 mL, once every 2 weeks)	100 cases: Intra-articular injection of compound betamethasone alone + oral diclofenac	Rasmussen score; VAS; total effective rate; adverse reactions
Shaohui Shi 2017 [36]	*n* = 286	159 cases: Intra-articular injection of medical chitosan (2 mL at 0, 2, 4 weeks) + oral glucosamine sulfate	127 cases: Intra-articular injection of sodium hyaluronate + oral glucosamine sulfate	VAS; Lysholm score
Tao Zeng 2018 [37]	*n* = 60	30 cases: Medical chitosan alone	30 cases: Weifeng powder alone	VAS; WOMAC; total effective rate
Lihui Ma 2021 [38]	*n* = 106	53 cases: Intra-articular injection of medical chitosan (2 mL, once every 2 weeks, 3 times)	53 cases: Intra-articular sodium hyaluronate + oral diclofenac	VAS; Lequesne; total effective rate
Zhenlong Bai 2015 [39]	*n* = 34	17 cases: Intra-articular injection of medical chitosan (2 mL, once every 2 weeks, 3 times)	17 cases: Intra-articular sodium hyaluronate (2 mL, once a week, 5 times)	VAS; WOMAC; subject global assessment; adverse reactions
Qi-Hang Su2024 [40]	*n* = 60	30 cases: Course-based intra-articular injection of medical chitosan (2 mL, 1–2 times/month)	30 cases: No pharmacological intervention	VAS; WOMAC; AKS; adverse events

Notes: Intervention group received intra-articular medical chitosan alone or combined with other therapies such as lidocaine, exercise, ozone, or oral drugs. Control group received conventional treatments without chitosan, for example NSAIDs, corticosteroids, hyaluronic acid, physical therapy, ozone, herbal wash, or health education only.

## Data Availability

No new data were created or analyzed in this study. All data supporting the findings are derived from previously published articles, as cited and referenced in the manuscript.

## Supplementary Materials

The following supporting information can be downloaded at: https://www.mdpi.com/article/10.3390/diseases14070252/s1, Supplementary File S1. Full search strategies for all databases; Supplementary Figure S1. Leave one out sensitivity analysis forest plots for VAS. Supplementary Figure S2. Leave one out sensitivity analysis forest plots for clinical efficacy. Supplementary Figure S3. Funnel plot for the meta-analysis of VAS scores. Supplementary Figure S4. Funnel plot for the meta-analysis of clinical efficacy. Supplementary Table S1. Evidence certainty and GRADE summary for clinical efficacy, VAS, Lequesne index, and WOMAC scores. Supplementary Table S2. Summary of findings and GRADE evidence profile for clinical efficacy, VAS, Lequesne index, and WOMAC scores.

## References

[1] Yang G., Wang J., Liu Y., Lu H., He L., Ma C., Zhao Z. (2023). Burden of Knee Osteoarthritis in 204 Countries and Territories, 1990–2019: Results From the Global Burden of Disease Study 2019. Arthritis Care Res..

[2] Neogi T. (2013). The epidemiology and impact of pain in osteoarthritis. Osteoarthr. Cartil..

[3] Li E., Tan J., Xu K., Pan Y., Xu P. (2024). Global burden and socioeconomic impact of knee osteoarthritis: A comprehensive analysis. Front. Med..

[4] Cross M., Smith E., Hoy D., Nolte S., Ackerman I., Fransen M., Bridgett L., Williams S., Guillemin F., Hill C.L. (2014). The global burden of hip and knee osteoarthritis: Estimates from the global burden of disease 2010 study. Ann. Rheum. Dis..

[5] Bannuru R.R., Osani M.C., Vaysbrot E.E., Arden N.K., Bennell K., Bierma-Zeinstra S.M.A., Kraus V.B., Lohmander L.S., Abbott J.H., Bhandari M. (2019). OARSI guidelines for the non-surgical management of knee, hip, and polyarticular osteoarthritis. Osteoarthr. Cartil..

[6] Roddy E., Zhang W., Doherty M. (2005). Aerobic walking or strengthening exercise for osteoarthritis of the knee? A systematic review. Ann. Rheum. Dis..

[7] Mo L., Jiang B., Mei T., Zhou D. (2023). Exercise Therapy for Knee Osteoarthritis: A Systematic Review and Network Meta-analysis. Orthop. J. Sports Med..

[8] Ozeki N., Koga H., Sekiya I. (2022). Degenerative Meniscus in Knee Osteoarthritis: From Pathology to Treatment. Life.

[9] Loeser R.F., Goldring S.R., Scanzello C.R., Goldring M.B. (2012). Osteoarthritis: A disease of the joint as an organ. Arthritis Rheum..

[10] Bruyère O., Cooper C., Pelletier J.P., Branco J., Luisa Brandi M., Guillemin F., Hochberg M.C., Kanis J.A., Kvien T.K., Martel-Pelletier J. (2014). An algorithm recommendation for the management of knee osteoarthritis in Europe and internationally: A report from a task force of the European Society for Clinical and Economic Aspects of Osteoporosis and Osteoarthritis (ESCEO). Semin. Arthritis Rheum..

[11] Hochberg M.C., Altman R.D., April K.T., Benkhalti M., Guyatt G., McGowan J., Towheed T., Welch V., Wells G., Tugwell P. (2012). American College of Rheumatology 2012 recommendations for the use of nonpharmacologic and pharmacologic therapies in osteoarthritis of the hand, hip, and knee. Arthritis Care Res..

[12] McAlindon T.E., Bannuru R.R., Sullivan M.C., Arden N.K., Berenbaum F., Bierma-Zeinstra S.M., Hawker G.A., Henrotin Y., Hunter D.J., Kawaguchi H. (2014). OARSI guidelines for the non-surgical management of knee osteoarthritis. Osteoarthr. Cartil..

[13] Picos-Corrales L.A., Morales-Burgos A.M., Ruelas-Leyva J.P., Crini G., García-Armenta E., Jimenez-Lam S.A., Ayón-Reyna L.E., Rocha-Alonzo F., Calderón-Zamora L., Osuna-Martínez U. (2023). Chitosan as an Outstanding Polysaccharide Improving Health-Commodities of Humans and Environmental Protection. Polymers.

[14] Garcia Garcia C.E., Lardy B., Bossard F., Soltero Martínez F.A., Rinaudo M. (2021). Chitosan based biomaterials for cartilage tissue engineering: Chondrocyte adhesion and proliferation. Food Hydrocoll. Health.

[15] Lynen N.A., Eichhorn C., Portelange N., Chausson M., Weyenberg W. (2024). Long-Term Efficacy Following Intra-articular Injection of Carboxymethyl-chitosan, a New Product Class for Knee Osteoarthritis: Results from an Observational Study in Germany. Rheumatol. Ther..

[16] Manocchio N., Ljoka C., Piacentini N., Sorge R., Vita G., Foti C. (2024). Intra-articular injections with Carboxymethyl-Chitosan in patients affected by knee osteoarthritis non-responders to hyaluronic acid: A pilot study. Eur. J. Transl. Myol..

[17] Manocchio N., Pirri C., Ljoka C., Sorbino A., Piacentini N., Monello C., Vita G., Foti C. (2025). Long-Term Efficacy of Carboxymethyl-Chitosan in Advanced Knee Osteoarthritis: A Twelve-Month Follow-Up Study on Non-Responders to Hyaluronic Acid. Biomedicines.

[18] Emans P., Skaliczki G., Haverkamp D., Bentin J., Chausson M., Schifflers M., Hermitte L., Douette P. (2022). First-in-human study to evaluate a single injection of KiOmedine^®^ CM-Chitosan for treating symptomatic knee osteoarthritis. Open Rheumatol. J..

[19] Novy T.C.T., Joni I.M., Lesmana R., Biben V., Setiawan (2025). Chitosan Nanoparticles as an Alternative Therapeutic Approach for Knee Osteoarthritis Treatment: A Systematic Review. Int. J. Nanomed..

[20] Ravindranathan S., Koppolu B.P., Smith S.G., Zaharoff D.A. (2016). Effect of Chitosan Properties on Immunoreactivity. Mar. Drugs.

[21] McAlindon T.E., LaValley M.P., Gulin J.P., Felson D.T. (2000). Glucosamine and chondroitin for treatment of osteoarthritis: A systematic quality assessment and meta-analysis. JAMA.

[22] Zhang W., Doherty M., Arden N., Bannwarth B., Bijlsma J., Gunther K.P., Hauselmann H.J., Herrero-Beaumont G., Jordan K., Kaklamanis P. (2005). EULAR evidence based recommendations for the management of hip osteoarthritis: Report of a task force of the EULAR Standing Committee for International Clinical Studies Including Therapeutics (ESCISIT). Ann. Rheum. Dis..

[23] Cui A., Li H., Wang D., Zhong J., Chen Y., Lu H. (2020). Global, regional prevalence, incidence and risk factors of knee osteoarthritis in population-based studies. eClinicalMedicine.

[24] Bedenbaugh A.V., Bonafede M., Marchlewicz E.H., Lee V., Tambiah J. (2021). Real-World Health Care Resource Utilization and Costs Among US Patients with Knee Osteoarthritis Compared with Controls. Clin. Outcomes Res..

[25] Smith T.O., Hawker G.A., Hunter D.J., March L.M., Boers M., Shea B.J., Christensen R., Guillemin F., Terwee C.B., Williamson P.R. (2019). The OMERACT-OARSI Core Domain Set for Measurement in Clinical Trials of Hip and/or Knee Osteoarthritis. J. Rheumatol..

[26] Page M.J., McKenzie J.E., Bossuyt P.M., Boutron I., Hoffmann T.C., Mulrow C.D., Shamseer L., Tetzlaff J.M., Akl E.A., Brennan S.E. (2021). The PRISMA 2020 statement: An updated guideline for reporting systematic reviews. BMJ.

[27] Shao N., Hu L. (2020). Analysis of the efficacy of intra-articular injection of medical chitosan for the treatment of knee osteoarthritis. China Pract. Med..

[28] Li P. (2022). Analysis of the Effect of Intra-Articular Injection of Medical Chitosan in the Treatment of Knee Osteoarthritis. Heal Friend.

[29] Chen J., Tian D. (2021). A Study on the Efficacy of Intra-articular Injection of Medical Chitosan + Lidocaine in the Treatment of Knee Osteoarthritis. Contemp. Med. Forum.

[30] Jiang Y. (2024). Analysis of the Effect of Intra-Articular Injection of Medical Chitosan Combined with Exercise Prescription on Patients with Moderate Knee Osteoarthritis. Chin. Sci. Technol. Period. Database Med..

[31] Li S., Li Z., Deng Q., Sang X., Zhou G., Zhang H. (2021). Effect of Three-dimensional Health Therapy on knee osteoarthritis. Hebei J. Tradit. Chin. Med..

[32] Sun C., Zhang R. (2022). Clinical Efficacy of Acupotomy Release Combined with Intra-Articular Dual Injection of Ozone and Medical Chitosan in the Treatment of Senile Knee Osteoarthritis. Chin. J. Gerontol..

[33] Jin Y.E., Yunzhou Z.U.O., Fan F.A.N., Xiaokang Y.A.N., Changjun Z.U.O., Xianzhong M.E.I., Qi Z. (2019). Research on curative effects of medical chitosan combined with diacerein in treatment of knee osteoarthritis. China Med. Pharm..

[34] Chen X. (2015). Observation on the Efficacy of Ozone Irrigation Combined with Injection of Medical Chitosan in the Treatment of Knee Osteoarthritis. J. Minim. Invasive Med..

[35] Li Z., Dai H., Li Y. (2021). Investigation on the Clinical Efficacy of Intra-Articular Injection of Compound Betamethasone Combined with Medical Chitosan in the Treatment of Knee Osteoarthritis. J. Front. Med..

[36] Shi S., Zhang P., Wu G., Zhen Y. (2017). Clinical analysis of complications for elderly patients with severe knee arthritis after intra-articular injection. Chin. J. Clin. (Electron. Ed.).

[37] Zeng T., Tang J., Gao D., Chen L. (2018). Clinical Observation on Weifeng Guke External Washing Powder Combined with Articular Cavity Injection of Medical Chitosan for Knee Osteoarthritis. J. New Chin. Med..

[38] Ma L. (2021). Analysis of the Efficacy of Intra-Articular Injection of Medical Chitosan in the Treatment of Knee Osteoarthritis. Healthmust-Readmagazine.

[39] Bai Z., He Y. (2015). Comparison of therapeutic effect of chitosan and sodium hyaluronate intra-articular injection in the treatment of knee osteoarthritis. Surg. Res. New Tech..

[40] Su Q.-H., Chen L.-Y., Cai Q.-C., Ge H.-A., Li J., Liu C.-T., Xue C., Huang J.-B., Huang C.-L., Feng X.-F. (2024). Course-based intra-articular injection of medical chitosan mitigates excessive deposition of triacylglycerides in the synovial tissue of the knee osteoarthritis. J. Chin. Med. Assoc..

[41] Cumpston M., Li T., Page M.J., Chandler J., Welch V.A., Higgins J.P., Thomas J. (2019). Updated guidance for trusted systematic reviews: A new edition of the Cochrane Handbook for Systematic Reviews of Interventions. Cochrane Database Syst. Rev..

[42] Di Martino A., Di Matteo B., Papio T., Tentoni F., Selleri F., Cenacchi A., Kon E., Filardo G. (2019). Platelet-Rich Plasma Versus Hyaluronic Acid Injections for the Treatment of Knee Osteoarthritis: Results at 5 Years of a Double-Blind, Randomized Controlled Trial. Am. J. Sports Med..

[43] Cao R., Yu H., Long H., Zhang H., Hao C., Shi L., Du Y., Jiao S., Guo A., Ma L. (2022). Low deacetylation degree chitosan oligosaccharide protects against IL-1β induced inflammation and enhances autophagy activity in human chondrocytes. J. Biomater. Sci. Polym. Ed..

[44] Schaible H.G., von Banchet G.S., Boettger M.K., Brauer R., Gajda M., Richter F., Hensellek S., Brenn D., Natura G. (2010). The role of proinflammatory cytokines in the generation and maintenance of joint pain. Ann. N. Y Acad. Sci..

[45] Swärd P., Frobell R., Englund M., Roos H., Struglics A. (2012). Cartilage and bone markers and inflammatory cytokines are increased in synovial fluid in the acute phase of knee injury (hemarthrosis)—A cross-sectional analysis. Osteoarthr. Cartil..

[46] Chakrabarti S., Jadon D.R., Bulmer D.C., Smith E.S.J. (2020). Human osteoarthritic synovial fluid increases excitability of mouse dorsal root ganglion sensory neurons: An in-vitro translational model to study arthritic pain. Rheumatology.

[47] Chen C., Yang F., Chen R., Yang C., Xiao H., Geng B., Xia Y. (2024). TRPV Channels in Osteoarthritis: A Comprehensive Review. Biomolecules.

[48] Karami P., Laurent A., Philippe V., Applegate L.A., Pioletti D.P., Martin R. (2024). Cartilage Repair: Promise of Adhesive Orthopedic Hydrogels. Int. J. Mol. Sci..

[49] Wan H., Ren K., Kaper H.J., Sharma P.K. (2020). A bioinspired mucoadhesive restores lubrication of degraded cartilage through reestablishment of lamina splendens. Colloids Surf. B Biointerfaces.

[50] Ata E., Özgüç S., Temel M.H., Beyaztaş H., Aktaş S., Güler E.M. (2024). The impact of ex vivo ozone injection into the synovial fluid in patients with knee osteoarthritis: A controlled clinical trial. Arch. Rheumatol..

[51] Li X., Dong Y., Liu J., He W., Yan C., Zhang J. (2025). Efficacy of arthroscopic cartilage transplantation combined with platelet-rich plasma in the treatment of early knee osteoarthritis: A retrospective cohort study. Langenbecks Arch. Surg..

[52] Emans P.J., Skaliczki G., Haverkamp D., Bentin J., Chausson M., Schifflers M., Portelange N. (2023). KiOmedine^®^ CM-Chitosan is Effective for Treating Advanced Symptomatic Knee Osteoarthritis up to Six Months Following a Single Intra-Articular Injection: A Post Hoc Analysis of Aproove Clinical Study. Open Rheumatol. J..

[53] Yu L., Wang T., Huang J., Zhang X. (2024). Efficacy of Intra-Articular Injection of Chitosan in Conjunction with Traditional Chinese Medicine Guidance Therapy in Knee Osteoarthritis. Indian. J. Pharm. Sci..

[54] Kunanusornchai W., Witoonpanich B., Tawonsawatruk T., Pichyangkura R., Chatsudthipong V., Muanprasat C. (2016). Chitosan oligosaccharide suppresses synovial inflammation via AMPK activation: An in vitro and in vivo study. Pharmacol. Res..

[55] Muzzarelli R.A., Greco F., Busilacchi A., Sollazzo V., Gigante A. (2012). Chitosan, hyaluronan and chondroitin sulfate in tissue engineering for cartilage regeneration: A review. Carbohydr. Polym..

[56] Deng J., Wei R., Qiu H., Wu X., Yang Y., Huang Z., Miao J., Liu A., Chai H., Cen X. (2024). Biomimetic zwitterionic copolymerized chitosan as an articular lubricant. Carbohydr. Polym..

[57] Pavelká K., Gatterová J., Olejarová M., Machacek S., Giacovelli G., Rovati L.C. (2002). Glucosamine sulfate use and delay of progression of knee osteoarthritis: A 3-year, randomized, placebo-controlled, double-blind study. Arch. Intern. Med..

[58] Raposo F., Ramos M., Lúcia Cruz A. (2021). Effects of exercise on knee osteoarthritis: A systematic review. Musculoskelet. Care.

[59] Rabea E.I., Badawy M.E., Stevens C.V., Smagghe G., Steurbaut W. (2003). Chitosan as antimicrobial agent: Applications and mode of action. Biomacromolecules.

[60] Kurita K. (2006). Chitin and Chitosan: Functional Biopolymers from Marine Crustaceans. Mar. Biotechnol..

[61] Moher D., Hopewell S., Schulz K.F., Montori V., Gotzsche P.C., Devereaux P.J., Elbourne D., Egger M., Altman D.G. (2010). CONSORT 2010 explanation and elaboration: Updated guidelines for reporting parallel group randomised trials. BMJ.

[62] Sterne J.A.C., Savovic J., Page M.J., Elbers R.G., Blencowe N.S., Boutron I., Cates C.J., Cheng H.Y., Corbett M.S., Eldridge S.M. (2019). RoB 2: A revised tool for assessing risk of bias in randomised trials. BMJ.

[63] Altman D.G., Schulz K.F., Moher D., Egger M., Davidoff F., Elbourne D., Gøtzsche P.C., Lang T. (2001). The revised CONSORT statement for reporting randomized trials: Explanation and elaboration. Ann. Intern. Med..

[64] Sterne J.A., Hernán M.A., Reeves B.C., Savović J., Berkman N.D., Viswanathan M., Henry D., Altman D.G., Ansari M.T., Boutron I. (2016). ROBINS-I: A tool for assessing risk of bias in non-randomised studies of interventions. BMJ.

