# Supplementary material for: Effect of Medical Chitosan on Clinical Efficacy and Pain in Knee Osteoarthritis: A Systematic Review and Meta-Analysis
Source: Diseases. 2026 Jul 14;14(7):252. doi: 10.3390/diseases14070252 (PMC13408866; doi:10.3390/diseases14070252)
Supplement: Supplementary file 1 [file diseases-14-00252-s001.zip › Supplementary File S1- Full search strategies for all databases -1.pdf]

Supplementary File S1- Full search strategies for all databases

1. Cochrane library search strategy

URL: https://www.cochranelibrary.com/

Accessed Date: 5 November 2024

#1(Knee Osteoarthritides OR KOA OR Knee Osteoarthritis OR Osteoarthritis of Knee OR Osteoarthritis of the Knee OR Osteoarthritis of the knee joint OR osteoarthritis of articular genu):ti,ab,kw

#2 MeSH descriptor: [knee osteoarthritis] explode all trees

#3 MeSH descriptor: [KOA] explode all trees

#4 #1 OR #2 OR #3

#5 MeSH descriptor: [chitosan] explode all trees

#6 (chitosan OR Poliglusam OR Medical-grade Chitosan OR Medical Chitin-derivative OR Medical Chitosan OR Chitosan for Medical Use OR Medical hexose OR Medical chitose):ti,ab,kw

#7 #5 OR #6

#8 MeSH descriptor: [Randomized Controlled Trial*] explode all trees

#9 (random allocation OR Controlled Clinical Trials as Topic OR control groups OR clinical trials as topic OR double-blind method OR single-blind method OR Placebos OR placebo effect OR cross-over studies OR Research Design OR randomized controlled trial ［pt］ OR controlled clinical trial ［pt］ OR clinical trial ［pt］):ti,ab,kw

#10 (random* or RCT or RCTs):ti,ab,kw

#11 (controlled AND (trial* or stud*)):ti,ab,kw

#12 (clinical* AND trial*):ti,ab,kw

#13 ((control OR treatment OR experiment* OR intervention) AND (group* OR subject* OR patient*)):ti,ab,kw

#14 (quasi-random* OR quasi random* OR pseudo-random* OR pseudo random*):ti,ab,kw

#15 ((control OR experiment* OR conservative) AND (treatment OR therapy OR procedure OR manage*)):ti,ab,kw

#16 ((singl* OR doubl* OR tripl* OR trebl*) AND (blind* or mask*)):ti,ab,kw

#17 (cross-over OR cross over OR crossover):ti,ab,kw

#18 (placebo* OR sham):ti,ab,kw

#19 trial [ti]

#20 (assign* OR allocate*):ti,ab,kw

#21 OR/#8-#20

#22 #4 AND #7 AND #21

2. PubMed search strategy

URL: https://pubmed.ncbi.nlm.nih.gov/

Accessed Date: 5 November 2024

#1 "Osteoarthritis, Knee"[Mesh]

#2 (Knee Osteoarthritides[Title/Abstract]) OR (Knee Osteoarthritis[Title/Abstract])) OR (Osteoarthritis of Knee[Title/Abstract])) OR (Osteoarthritis of the Knee[Title/Abstract])) OR (Osteoarthritis of the knee joint[Title/Abstract])) OR (osteoarthritis articular genu[Title/Abstract]

#3 #1 OR #2

#4 "Chitosan"[Mesh]

#5 (Poliglusam) OR (Medical-grade Chitosan)) OR (Medical Chitin-derivative)) OR (Medical chitose)) OR (Medical Chitosan)) OR (Chitosan for Medical Use)) OR (Medical hexose)

#6 #4 OR #5

#7 "Randomized Controlled Trials as Topic"[Mesh] OR "Placebos"[Mesh]

#8 Clinical Trials, Randomized[Title/Abstract] OR Trials, Randomized Clinical[Title/Abstract] OR Controlled Clinical Trials, Randomized[Title/Abstract] OR randomized controlled trial[Publication Type] OR placebo[Title/Abstract] OR randomized[Title/Abstract]

#9 #7 OR #8

#10 #3 AND #6 AND #9

((("Osteoarthritis, Knee"[Mesh]) OR ((((((Knee Osteoarthritides[Title/Abstract]) OR (Knee Osteoarthritis[Title/Abstract])) OR (Osteoarthritis of Knee[Title/Abstract])) OR (Osteoarthritis of the Knee[Title/Abstract])) OR (Osteoarthritis of the knee joint[Title/Abstract])) OR (osteoarthritis articular genu[Title/Abstract]))) AND (("Chitosan"[Mesh]) OR (((((((Poliglusam) OR (Medical-grade Chitosan)) OR (Medical Chitin-derivative)) OR (Medical chitose)) OR (Medical Chitosan)) OR (Chitosan for Medical Use)) OR (Medical hexose)))) AND ((("Randomized Controlled Trials as Topic"[Mesh]) OR ("Placebos"[Mesh])) OR (((((((Clinical Trials, Randomized[Title/Abstract]) OR (Trials, Randomized Clinical[Title/Abstract])) OR (Controlled Clinical Trials, Randomized[Title/Abstract])) OR (randomized controlled trial[Publication Type]))) OR (placebo[Title/Abstract])) OR (randomized[Title/Abstract])))

3. Web Of Science search strategy

URL: https://www.webofscience.com/

Accessed Date: 5 November 2024

#1 TS=(knee osteoarthritis OR KOA OR Knee Osteoarthritides OR Knee Osteoarthritis OR Osteoarthritis of Knee OR Osteoarthritis of the Knee OR Osteoarthritis of the knee joint OR osteoarthritis articular genu)

#2 TS=(chitosan OR Poliglusam OR Medical-grade Chitosan OR Medical Chitin-derivative OR Medical chitose OR Medical Chitosan OR Chitosan for Medical Use OR Medical hexose)

#3 TS=(Clinical Trials, Randomized OR Trials, Randomized Clinical OR Controlled Clinical Trials, Randomized OR randomized controlled trial[Publication Type] OR placebo OR randomized OR random* OR cross over* OR crossover* OR controlled placepo*)

#4 #1 AND #2 AND #3

((TS=(knee osteoarthritis OR KOA OR Knee Osteoarthritides OR Knee Osteoarthritis OR Osteoarthritis of Knee OR Osteoarthritis of the Knee OR Osteoarthritis of the knee joint OR osteoarthritis articular genu)) AND TS=(chitosan OR Poliglusam OR Medical-grade Chitosan OR Medical Chitin-derivative OR Medical chitose OR Medical Chitosan OR Chitosan for Medical Use OR Medical hexose)) AND TS=(Clinical Trials, Randomized OR Trials, Randomized Clinical OR Controlled Clinical Trials, Randomized OR randomized controlled trial[Publication Type] OR placebo OR randomized OR random* OR cross over* OR crossover* OR controlled placepo*)

4. EBSCO search strategy

URL: https://www.ebsco.com/

Accessed Date: 5 November 2024

#1
((knee osteoarthritis or KOA or Knee Osteoarthritides or Knee Osteoarthritis or Osteoarthritis of Knee or Osteoarthritis of the Knee or Osteoarthritis of the knee joint or osteoarthritis articular genu)) OR AB ((knee osteoarthritis or KOA or Knee Osteoarthritides or Knee Osteoarthritis or Osteoarthritis of Knee or Osteoarthritis of the Knee or Osteoarthritis of the knee joint or osteoarthritis articular genu))

#2
((chitosan OR Poliglusam OR Medical-grade Chitosan OR Medical Chitin-derivative OR Medical chitose OR Medical Chitosan OR Chitosan for Medical Use OR Medical hexose)) OR AB ((chitosan OR Poliglusam OR Medical-grade Chitosan OR Medical Chitin-derivative OR Medical chitose OR Medical Chitosan OR Chitosan for Medical Use OR Medical hexose))

#3
((Clinical Trials, Randomized or Trials, Randomized Clinical or Controlled Clinical Trials, Randomized or randomized controlled trial[Publication Type] or placebo or randomized or random* or cross over* or crossover* or controlled placepo*)) OR AB ((Clinical Trials, Randomized or Trials, Randomized Clinical or Controlled Clinical Trials, Randomized or randomized controlled trial[Publication Type] or placebo or randomized or random* or cross over* or crossover* or controlled placepo*))

#4 #1 AND #2 AND #3

5. PsycINFO search strategy

URL: https://www.apa.org/psycinfo/

Accessed Date: 5 November 2024

#1 (knee osteoarthritis or KOA or Knee Osteoarthritides or Knee Osteoarthritis or Osteoarthritis of Knee or Osteoarthritis of the Knee or Osteoarthritis of the knee joint or osteoarthritis articular genu) OR AB(knee osteoarthritis or KOA or Knee Osteoarthritides or Knee Osteoarthritis or Osteoarthritis of Knee or Osteoarthritis of the Knee or Osteoarthritis of the knee joint or osteoarthritis articular genu)

#2 (chitosan OR Poliglusam OR Medical-grade Chitosan OR Medical Chitin-derivative OR Medical chitose OR Medical Chitosan OR Chitosan for Medical Use OR Medical hexose) OR AB (chitosan OR Poliglusam OR Medical-grade Chitosan OR Medical Chitin-derivative OR Medical chitose OR Medical Chitosan OR Chitosan for Medical Use OR Medical hexose)

#3 (Clinical Trials, Randomized or Trials, Randomized Clinical or Controlled Clinical Trials, Randomized or randomized controlled trial[Publication Type] or placebo or randomized or random* or cross over* or crossover* or controlled placepo*) OR AB (Clinical Trials, Randomized or Trials, Randomized Clinical or Controlled Clinical Trials, Randomized or randomized controlled trial[Publication Type] or placebo or randomized or random* or cross over* or crossover* or controlled placepo*)

#4 (double-blind ［tw］ OR random: asigned ［tw］ OR control ［tw］) OR AB (double-blind ［tw］ OR random: asigned ［tw］ OR control ［tw］)

#5 #3 OR #4

#6 #1 AND #2 AND #5

6. Clinical trials search strategy

URL: https://clinicaltrials.gov/

Accessed Date: 5 November 2024

#1 Condition/disease (Clinical Trials, Randomized OR Trials, Randomized Clinical OR Controlled Clinical Trials, Randomized)

#2 Other terms (knee osteoarthritis OR KOA OR Knee Osteoarthritides OR Knee Osteoarthritis OR Osteoarthritis of Knee OR Osteoarthritis of the Knee OR Osteoarthritis of the knee joint OR osteoarthritis articular genu)

#3 Intervention/treatment (chitosan OR Poliglusam OR Medical-grade Chitosan OR Medical Chitin-derivative OR Medical chitose OR Medical Chitosan OR Chitosan for Medical Use OR Medical hexose)

7. Sinomed search strategy

URL: https://www.sinomed.ac.cn/

Accessed Date: 5 November 2024

((("medical chitosan"[Common Fields: Intelligent] OR "medical chitin"[Common Fields: Intelligent]) OR "chitosan-based medical products"[Common Fields: Intelligent] OR "medical chitosan"[Common Fields: Intelligent] OR "chitosan derivative medical preparations"[Common Fields: Intelligent]) AND ("knee osteoarthritis"[Common Fields: Intelligent] OR "knee arthritis"[Common Fields: Intelligent] OR "knee osteoarthrosis"[Common Fields: Intelligent] OR "knee degenerative joint disease"[Common Fields: Intelligent] OR "knee degenerative arthritis"[Common Fields: Intelligent] OR "knee osteoarthritis"[Common Fields: Intelligent]))

8.Chinese Medical Journal Network search strategy

URL: https://www.medjournals.cn/

Accessed Date: 5 November 2024

("medical chitosan" OR "medical chitin" OR "chitosan-based medical products" OR "medical chitosan" OR "chitosan derivative medical preparations") AND ("knee osteoarthritis" OR "knee osteoarthrosis" OR "knee degenerative joint disease" OR "knee degenerative arthritis" OR "knee arthritis" OR "knee osteoarthritis")

9. CNKI search strategy

URL: https://www.cnki.net/

Accessed Date: 5 November 2024

#1 (Subject = "knee osteoarthritis" OR "knee osteoarthritis (OA)" OR "knee osteoarthritis pain" OR "knee osteoarthritis score" OR "knee osteoarthritis patient")

#2 (Subject = "medical chitosan" OR "medical chitin" OR "chitosan-based medical products" OR "medical chitosan" OR "chitosan derivative medical preparations")

10. VIP search strategy

URL: http://www.cqvip.com/

Accessed Date: 5 November 2024

(Title/Keyword = "knee arthritis" OR "knee osteoarthritis" OR "knee osteoarthrosis" OR "knee degenerative joint disease" OR "knee degenerative arthritis" OR "knee arthritis" OR "knee osteoarthritis")
AND
(Title/Keyword = "medical chitosan" OR "medical chitin" OR "chitosan-based medical products" OR "medical chitosan" OR "chitosan derivative medical preparations")

11. Wanfang search strategy

URL: https://www.wanfangdata.com.cn/

Accessed Date: 5 November 2024

(Subject:("knee arthritis") OR Title/Keyword:("knee osteoarthritis" OR "knee degenerative arthritis" OR "knee Bi syndrome" OR "knee degenerative joint disease" OR "knee osteoarthrosis" OR "knee osteoarthritis"))
AND
(Subject:("medical chitosan") OR Title/Keyword:("medical chitin" OR "chitosan-based medical products" OR "medical chitosan" OR "chitosan derivative medical preparations"))

12. Embase search strategy

URL: https://www.embase.com/

Accessed Date: 5 November 2024

#1 ('osteoarthritis,'/exp OR osteoarthritis,) AND ('knee'/exp OR knee)

#2 'Knee Osteoarthritides' OR 'Knee Osteoarthritis' OR 'Osteoarthritis of Knee' OR 'Osteoarthritis of the Knee' OR 'Osteoarthritis of the knee joint' OR 'osteoarthritis articular genu'

#3 #1 OR #2

#4 chitosan

#5 'Poliglusam' OR 'Medical-grade Chitosan' OR 'Medical Chitin-derivative' OR 'Medical chitose' OR 'Medical Chitosan' OR 'Chitosan for Medical Use' OR 'Medical hexose'

#6 #4 OR #5

#7 'random【tw】' OR 'placebo【mp】' OR 'double blind【tw】'

#8 'Clinical Trials, Randomized' OR 'Trials, Randomized Clinical' OR 'Controlled Clinical Trials, Randomized' OR 'randomized controlled trial[Publication Type]' OR 'placebo' OR 'randomized'

#9 #7 OR #8

#10 #3 AND #6 AND #9

13. Scopus search strategy

URL: https://www.scopus.com/

Accessed Date: 5 November 2024

#1 TITLE-ABS-KEY(“knee osteoarthritis”)

#2 TITLE-ABS-KEY(”Knee Osteoarthritides”) OR TITLE-ABS-KEY(“Knee Osteoarthritis”) OR TITLE-ABS-KEY(“Osteoarthritis of Knee”) OR TITLE-ABS-KEY(“Osteoarthritis of the Knee”) OR TITLE-ABS-KEY(“Osteoarthritis of the knee joint”) OR TITLE-ABS-KEY(“osteoarthritis articular genu”)

#3 TITLE-ABS-KEY(“chitosan”)

#4 TITLE-ABS-KEY(“Poliglusam”) OR TITLE-ABS-KEY(“Medical-grade Chitosan”) OR TITLE-ABS-KEY(“Medical Chitin-derivative”) OR TITLE-ABS-KEY(“Medical chitose”) OR TITLE-ABS-KEY(“Medical Chitosan”) OR TITLE-ABS-KEY(“Chitosan for Medical Use”) OR TITLE-ABS-KEY(“Medical hexose”)

#5 TITLE-ABS-KEY ("Randomized Controlled Trials as Topic") OR TITLE-ABS-KEY ("Placebos")

#6 TITLE-ABS-KEY(“Clinical Trials, Randomized”) OR TITLE-ABS-KEY(“Trials, Randomized Clinical”) OR TITLE-ABS-KEY(“Controlled Clinical Trials, Randomized”) OR TITLE-ABS-KEY(“randomized controlled trial[Publication Type]”) OR TITLE-ABS-KEY(“placebo”) OR TITLE-ABS-KEY(“randomized”)

#7 #1 OR #2

#8 #3 OR #4

#9 #5 OR #6

#10 #7 AND #8 AND #9
