# Supplementary material for: Effect of Medical Chitosan on Clinical Efficacy and Pain in Knee Osteoarthritis: A Systematic Review and Meta-Analysis
Source: Diseases. 2026 Jul 14;14(7):252. doi: 10.3390/diseases14070252 (PMC13408866; doi:10.3390/diseases14070252)
Supplement: Supplementary file 1 [file diseases-14-00252-s001.zip › Supplementary Table S1. Evidence certainty and GRADE summary for clinical efficacy, VAS, Lequesne index, and WOMAC scores.pdf]

Supplementary Table S1. Evidence certainty and GRADE summary for clinical efficacy, VAS, Lequesne index, and WOMAC scores.

Certainty assessment							Summary of findings				
Participants (studies) and follow-up	Risk of bias	Inconsistency	Indirectness	Imprecision	Publication bias	Overall certainty of evidence	Study event rate (%)		Relative effect (95% CI)	Anticipated absolute effect	
							with [control]	with [intervention]		Risk with [control]	Risk difference with [intervention]
**Clinical efficacy (total effective rate)**											
1052 (10 RCTs)	**Serious**[a]	**Not serious**	**Not serious**	**Not serious**	**None**	⊕⊕⊕◯ Moderate[a]	443/526 (84.2%)	509/526 (96.8%)	**比值比 5.43** (3.21 到 9.18)	443/526 (84.2%)	124 more per 1,000 (from 103 more to 138 more)
**VAS pain score**											
1378 (12 RCTs)	**Serious**[b]	**Very serious**[c]	**Not serious**	**Not serious**	**None**	⊕◯◯◯ Very low[b,c]	705	673	-	705	MD −1.06 (−1.38 to −0.73)
**Lequesne index**											
442 (4 RCTs)	**Serious**[d]	**Very serious**[e]	**Not serious**	**Serious**[f]	**None**	⊕◯◯◯ Very low[d,e,f]	220	222	-	220	MD −3.16 (−5.39 to −0.92)
**WOMAC score**											
298 (5 RCTs)	**Serious**[g]	**Very serious**[h]	**Not serious**	**Serious**[i]	**None**	⊕◯◯◯ Very low[g,h,i]	149	149	-	149	MD −4.24 (−6.22 to −2.26)

CI: Confidence interval; **MD:** Mean difference; **OR:** Odds ratio

Explanations

a. All 10 enrolled randomized controlled trials were rated high overall risk of bias in Cochrane RoB2 evaluation. None of studies reported detailed random sequence generation methods or allocation concealment measures. No trial implemented blinding for participants, clinicians or outcome assessors. The definition of clinical effective rate relied on self-formulated composite clinical criteria without unified objective standard; lack of blinding would lead to subjective overestimation of treatment response, resulting in serious risk of bias across all included studies.

b. All 12 included RCTs had high overall risk of bias. None described complete random sequence generation or allocation concealment, and no trial adopted any blinding for participants, clinicians or outcome assessors. VAS is patient-reported subjective pain scale; unblinded design easily triggers subjective measurement bias and overestimates analgesic efficacy of experimental therapy, leading to serious risk of bias across all studies.

c. Heterogeneity statistics: $\chi^2=600.46$, df=11, $P<0.00001$, $I^2=98\%>75\%$, which represents extreme statistical inconsistency. Major reasons for extreme heterogeneity: inconsistent chitosan dosage, administration frequency, combined auxiliary treatments, varying K-L grades of enrolled KOA patients and different follow-up durations among trials; subgroup analysis cannot explain such enormous between-study fluctuation, so inconsistency is rated very serious for another downgrade.

d. All four included RCTs had high overall risk of bias. None of the trials fully reported random sequence generation and allocation concealment details, and no study implemented blinding for participants, clinicians or outcome assessors. Lequesne index is a subjective patient-reported functional assessment scale; absence of blinding leads to subjective overestimation of joint function improvement of experimental group, which causes serious risk of bias across all studies.

e. Extreme inter-study heterogeneity was observed: $\chi^2=302.56$, df=3, $P<0.00001$, $I^2=99\%>75\%$, defined as very serious inconsistency. The prominent heterogeneity originates from distinct differences in chitosan administration dose, combined treatment regimens, baseline K-L radiographic grading of enrolled KOA patients and follow-up duration among four trials; preset subgroup analysis cannot interpret such extreme variation between studies, so we downgrade another level for very serious inconsistency.

f. Total sample size is only 442 participants (222 intervention vs 220 control), merely four included trials. The pooled 95%CI [-5.39, -0.92] spans a very wide range of clinical effect from large clinical benefit down to mild minimal benefit; total sample quantity fails to reach optimal information size, existing substantial random uncertainty for pooled effect, so imprecision is rated serious and downgrade one more level.

g. All five included RCTs were rated high overall risk of bias by Cochrane RoB2. None of trials reported complete random sequence generation and allocation concealment details; no study applied blinding for participants, clinicians or outcome assessors. WOMAC is patient-reported subjective functional scale, non-blinded design leads to subjective overrating of functional improvement of experimental treatment, which brings serious risk of bias across all studies.

h. Heterogeneity data: $\chi^2=37.28$, df=4, $P<0.00001$, $I^2=89\%>75\%$, indicating very serious inconsistency. The prominent heterogeneity derives from inconsistent chitosan dosage, combination medication types, varied baseline K-L grades of enrolled KOA patients and inconsistent follow-up cycles among included trials; subgroup analysis cannot fully explain such large between-study variation.

i. Total sample size only 298 cases (149 experimental vs149 control), just five small-sample RCTs. Pooled 95%CI [-10.59,-2.71] spans a wide range of clinical efficacy from large functional improvement to mild improvement; total sample fails to reach optimal information size, the wide confidence interval brings big uncertainty to pooled effect, imprecision is serious and downgrade another level.
