# Supplementary material for: Effect of Medical Chitosan on Clinical Efficacy and Pain in Knee Osteoarthritis: A Systematic Review and Meta-Analysis
Source: Diseases. 2026 Jul 14;14(7):252. doi: 10.3390/diseases14070252 (PMC13408866; doi:10.3390/diseases14070252)
Supplement: Supplementary file 1 [file diseases-14-00252-s001.zip › Supplementary Table S2. Summary of findings and GRADE evidence profile for clinical efficacy, VAS, Lequesne index, and WOMAC scores.pdf]

Supplementary Table S2. Summary of findings and GRADE evidence profile for clinical efficacy, VAS, Lequesne index, and WOMAC score.

Outcome	*Anticipated absolute effect (95% CI)**		Relative effect (95% CI)	No. of participants (studies)	Certainty of the evidence (GRADE)	Comments
	Risk with [control]	Risk with [intervention]				
Clinical efficacy (total effective rate)	842 per 1,000	**967 per 1,000 (945 to 980)**	**Odds ratio 5.43 (3.21 to 9.18)**	1052 (10 RCTs)	⨁⨁⨁◯ Moderate[a]	Compared with control group, medical chitosan significantly improved the clinical effective rate of knee osteoarthritis. Pooled OR=5.43 (95%CI:3.21~9.18). Evidence certainty downgraded one level only due to serious overall risk of bias across all included RCTs; no obvious inconsistency, indirectness or imprecision was observed.
VAS pain score	Mean total VAS score was 3.67	MD 1.06 lower (1.38 lower to 0.73 lower)	-	1378 (12 RCTs)	⨁◯◯◯ Very low[b,c]	Medical chitosan reduced VAS pain score by MD=-1.06 (95%CI:-1.38~-0.73). Evidence downgraded for two reasons: serious risk of bias in all trials and very serious inconsistency (I^2=98%); no indirectness, imprecision was acceptable.
Lequesne index	Mean Lequesne score was 6.32	MD 3.16 lower (5.39 lower to 0.92 lower)	-	442 (4 RCTs)	⨁◯◯◯ Very low[d,e,f]	Intervention decreased Lequesne functional score by MD=-3.16 (95%CI:-5.39~-0.92). Certainty downgraded three times: serious risk of bias, very serious inconsistency(I^2=99%), plus serious imprecision from small sample size.
WOMAC score	Mean WOMAC score was 52.82	MD 6.65 lower (10.59 lower to 2.71 lower)	-	298 (5 RCTs)	⨁◯◯◯ Very low[g,h,i]	Chitosan therapy lowered WOMAC score by MD=-6.65 (95%CI:-10.59~-2.71). Downgrade for serious risk of bias, very serious inconsistency(I^2=89%) and serious imprecision with limited included sample.

***The risk in the intervention group (and its 95% confidence interval) is based on the assumed risk in the control group and the relative effect of the intervention (and its 95% confidence interval).**

CI: Confidence interval; **MD:** Mean difference; **OR:** Odds ratio

GRADE Working Group grades of evidence
High certainty: we are very confident that the true effect lies close to that of the estimate of the effect.
Moderate certainty: we are moderately confident in the effect estimate: the true effect is likely to be close to the estimate of the effect, but there is a possibility that it is substantially different.
Low certainty: our confidence in the effect estimate is limited: the true effect may be substantially different from the estimate of the effect.
Very low certainty: we have very little confidence in the effect estimate: the true effect is likely to be substantially different from the estimate of effect.

Explanations

a. All 10 enrolled randomized controlled trials were rated high overall risk of bias in Cochrane RoB2 evaluation. None of studies reported detailed random sequence generation methods or allocation concealment measures. No trial implemented blinding for participants, clinicians or outcome assessors. The definition of clinical effective rate relied on self-formulated composite clinical criteria without unified objective standard; lack of blinding would lead to subjective overestimation of treatment response, resulting in serious risk of bias across all included studies.

b. All 12 included RCTs had high overall risk of bias. None described complete random sequence generation or allocation concealment, and no trial adopted any blinding for participants, clinicians or outcome assessors. VAS is patient-reported subjective pain scale; unblinded design easily triggers subjective measurement bias and overestimates analgesic efficacy of experimental therapy, leading to serious risk of bias across all studies.

c. Heterogeneity statistics: χ^2=600.46, df=11, $P < 0.00001$, I^2=98% > 75%, which represents extreme statistical inconsistency. Major reasons for extreme heterogeneity: inconsistent chitosan dosage, administration frequency, combined auxiliary treatments, varying K-L grades of enrolled KOA patients and different follow-up durations among trials; subgroup analysis cannot explain such enormous between-study fluctuation, so inconsistency is rated very serious for another downgrade.

d. All four included RCTs had high overall risk of bias. None of the trials fully reported random sequence generation and allocation concealment details, and no study implemented blinding for participants, clinicians or outcome assessors. Lequesne index is a subjective patient-reported functional assessment scale; absence of blinding leads to subjective overestimation of joint function improvement of experimental group, which causes serious risk of bias across all studies.

e. Extreme inter-study heterogeneity was observed: χ^2=302.56, df=3， P < 0.00001, I^2=99% > 75%, defined as very serious inconsistency. The prominent heterogeneity originates from distinct differences in chitosan administration dose, combined treatment regimens, baseline K-L radiographic grading of enrolled KOA patients and follow-up duration among four trials; preset subgroup analysis cannot interpret such extreme variation between studies, so we downgrade another level for very serious inconsistency.

f. Total sample size is only 442 participants (222 intervention vs 220 control), merely four included trials. The pooled 95%CI [-5.39, -0.92] spans a very wide range of clinical effect from large clinical benefit down to mild minimal benefit; total sample quantity fails to reach optimal information size, existing substantial random uncertainty for pooled effect, so imprecision is rated serious and downgrade one more level.

g. All five included RCTs were rated high overall risk of bias by Cochrane RoB2. None of trials reported complete random sequence generation and allocation concealment details; no study applied blinding for participants, clinicians or outcome assessors. WOMAC is patient-reported subjective functional scale, non-blinded design leads to subjective overrating of functional improvement of experimental treatment, which brings serious risk of bias across all studies.

h. Heterogeneity data: χ^2=37.28, df=4, P < 0.00001， I^2=89% > 75%, indicating very serious inconsistency. The prominent heterogeneity derives from inconsistent chitosan dosage, combination medication types, varied baseline K-L grades of enrolled KOA patients and inconsistent follow-up cycles among included trials; subgroup analysis cannot fully explain such large between-study variation.

i. Total sample size only 298 cases (149 experimental vs149 control), just five small-sample RCTs. Pooled 95%CI [-10.59,-2.71] spans a wide range of clinical efficacy from large functional improvement to mild improvement; total sample fails to reach optimal information size, the wide confidence interval brings big uncertainty to pooled effect, imprecision is serious and downgrade another level.
