# Supplementary material for: Effect of Medical Chitosan on Clinical Efficacy and Pain in Knee Osteoarthritis: A Systematic Review and Meta-Analysis
Source: Diseases. 2026 Jul 14;14(7):252. doi: 10.3390/diseases14070252 (PMC13408866; doi:10.3390/diseases14070252)
Supplement: Supplementary file 1 [file diseases-14-00252-s001.zip › Supplementary_Figure_S1_Leave_one_out sensitivity analysis forest plots for VAS.pdf]

Supplementary Figure S1. Leave-one-out sensitivity analysis forest plots for VAS.

Study or Subgroup	Experimental Mean	Experimental SD	Experimental Total	Control Mean	Control SD	Control Total	Weight	Mean Difference IV, Random, 95% CI
Changle Sun 2022	1.67	0.24	42	3.88	0.47	42	0.0%	-2.21 [-2.37, -2.05]
Jian Chen 2021 a	1.86	0.18	90	2.91	0.26	90	10.8%	-1.05 [-1.12, -0.98]
Lihui Ma 2021	1.26	0.27	53	3.01	0.32	53	10.7%	-1.75 [-1.86, -1.64]
Na Shao 2020	1.5	0.53	54	2.66	0.74	54	10.1%	-1.16 [-1.40, -0.92]
Peng Li 2022	2.35	0.32	37	2.59	0.44	37	10.4%	-0.24 [-0.42, -0.06]
Qi-Hang Su 2024	4.47	1.22	30	5.13	1.2	30	7.2%	-0.66 [-1.27, -0.05]
Sen Li 2021	2.51	0.37	30	3.02	0.25	30	10.5%	-0.51 [-0.67, -0.35]
Shaohui Shi 2017 a	4.3	3.2	159	5.8	3.2	127	6.2%	-1.50 [-2.25, -0.75]
Tao Zeng 2018	2.4	0.49	30	3.66	0.6	30	9.8%	-1.26 [-1.54, -0.98]
Xinyu Chen 2015 a	0.54	0.13	63	1.22	0.27	63	10.8%	-0.68 [-0.75, -0.61]
Zhenlong Bai 2015 a	2.29	1.89	17	3.29	2.34	17	2.9%	-1.00 [-2.43, 0.43]
Zhi Li 2021	3.51	0.56	100	4.19	0.54	100	10.6%	-0.68 [-0.83, -0.53]
**Total (95% CI)**			**663**			**631**	**100.0%**	**-0.93 [-1.22, -0.65]**

Heterogeneity: Tau² = 0.19; Chi² = 368.16, df = 10 (P < 0.00001); I² = 97%
Test for overall effect: Z = 6.50 (P < 0.00001)

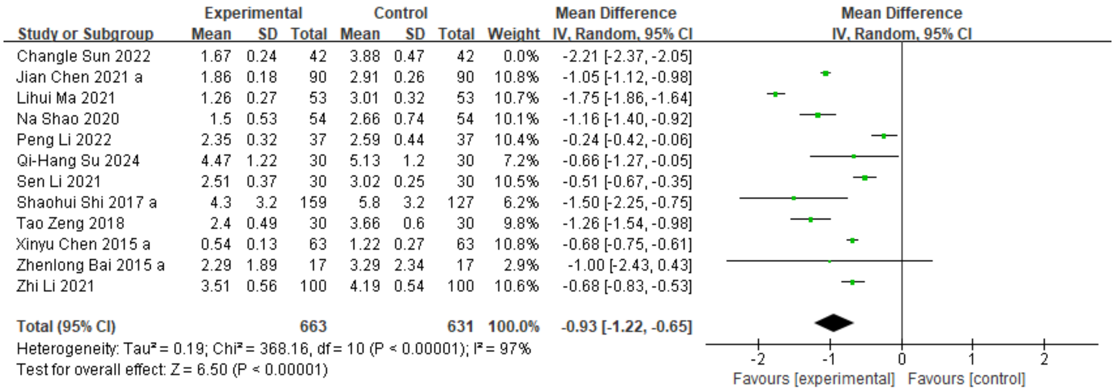

(a) Excluding Study A

Study or Subgroup	Experimental Mean	Experimental SD	Experimental Total	Control Mean	Control SD	Control Total	Weight	Mean Difference IV, Random, 95% CI
Changle Sun 2022	1.67	0.24	42	3.88	0.47	42	10.0%	-2.21 [-2.37, -2.05]
Jian Chen 2021 a	1.86	0.18	90	2.91	0.26	90	0.0%	-1.05 [-1.12, -0.98]
Lihui Ma 2021	1.26	0.27	53	3.01	0.32	53	10.1%	-1.75 [-1.86, -1.64]
Na Shao 2020	1.5	0.53	54	2.66	0.74	54	9.8%	-1.16 [-1.40, -0.92]
Peng Li 2022	2.35	0.32	37	2.59	0.44	37	9.9%	-0.24 [-0.42, -0.06]
Qi-Hang Su 2024	4.47	1.22	30	5.13	1.2	30	8.3%	-0.66 [-1.27, -0.05]
Sen Li 2021	2.51	0.37	30	3.02	0.25	30	10.0%	-0.51 [-0.67, -0.35]
Shaohui Shi 2017 a	4.3	3.2	159	5.8	3.2	127	7.6%	-1.50 [-2.25, -0.75]
Tao Zeng 2018	2.4	0.49	30	3.66	0.6	30	9.7%	-1.26 [-1.54, -0.98]
Xinyu Chen 2015 a	0.54	0.13	63	1.22	0.27	63	10.1%	-0.68 [-0.75, -0.61]
Zhenlong Bai 2015 a	2.29	1.89	17	3.29	2.34	17	4.6%	-1.00 [-2.43, 0.43]
Zhi Li 2021	3.51	0.56	100	4.19	0.54	100	10.0%	-0.68 [-0.83, -0.53]
**Total (95% CI)**			**615**			**583**	**100.0%**	**-1.06 [-1.47, -0.65]**

Heterogeneity: Tau² = 0.44; Chi² = 595.80, df = 10 (P < 0.00001); I² = 98%
Test for overall effect: Z = 5.02 (P < 0.00001)

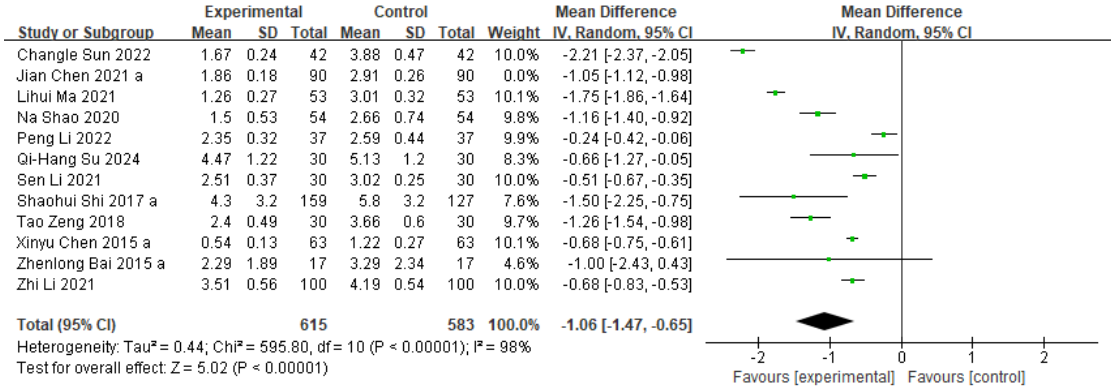

(b) Excluding Study B

Study or Subgroup	Experimental Mean	Experimental SD	Experimental Total	Control Mean	Control SD	Control Total	Weight	Mean Difference IV, Random, 95% CI
Changle Sun 2022	1.67	0.24	42	3.88	0.47	42	10.4%	-2.21 [-2.37, -2.05]
Jian Chen 2021 a	1.86	0.18	90	2.91	0.26	90	10.6%	-1.05 [-1.12, -0.98]
Lihui Ma 2021	1.26	0.27	53	3.01	0.32	53	0.0%	-1.75 [-1.86, -1.64]
Na Shao 2020	1.5	0.53	54	2.66	0.74	54	10.0%	-1.16 [-1.40, -0.92]
Peng Li 2022	2.35	0.32	37	2.59	0.44	37	10.3%	-0.24 [-0.42, -0.06]
Qi-Hang Su 2024	4.47	1.22	30	5.13	1.2	30	7.5%	-0.66 [-1.27, -0.05]
Sen Li 2021	2.51	0.37	30	3.02	0.25	30	10.4%	-0.51 [-0.67, -0.35]
Shaohui Shi 2017 a	4.3	3.2	159	5.8	3.2	127	6.6%	-1.50 [-2.25, -0.75]
Tao Zeng 2018	2.4	0.49	30	3.66	0.6	30	9.8%	-1.26 [-1.54, -0.98]
Xinyu Chen 2015 a	0.54	0.13	63	1.22	0.27	63	10.6%	-0.68 [-0.75, -0.61]
Zhenlong Bai 2015 a	2.29	1.89	17	3.29	2.34	17	3.3%	-1.00 [-2.43, 0.43]
Zhi Li 2021	3.51	0.56	100	4.19	0.54	100	10.4%	-0.68 [-0.83, -0.53]
**Total (95% CI)**			**652**			**620**	**100.0%**	**-0.98 [-1.29, -0.67]**

Heterogeneity: Tau² = 0.23; Chi² = 411.71, df = 10 (P < 0.00001); I² = 98%
Test for overall effect: Z = 6.24 (P < 0.00001)

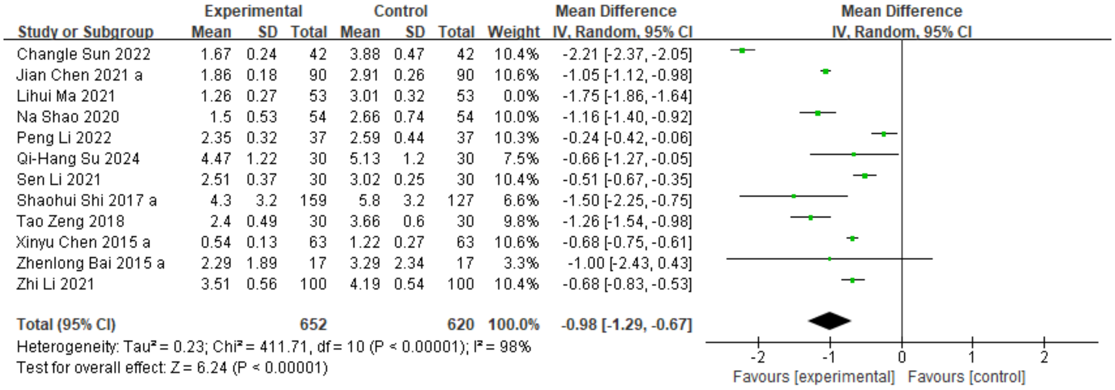

(c) Excluding Study C

Study or Subgroup	Experimental Mean	Experimental SD	Experimental Total	Control Mean	Control SD	Control Total	Weight	Mean Difference IV, Random, 95% CI
Changle Sun 2022	1.67	0.24	42	3.88	0.47	42	10.2%	-2.21 [-2.37, -2.05]
Jian Chen 2021 a	1.86	0.18	90	2.91	0.26	90	10.4%	-1.05 [-1.12, -0.98]
Lihui Ma 2021	1.26	0.27	53	3.01	0.32	53	10.3%	-1.75 [-1.86, -1.64]
Na Shao 2020	1.5	0.53	54	2.66	0.74	54	0.0%	-1.16 [-1.40, -0.92]
Peng Li 2022	2.35	0.32	37	2.59	0.44	37	10.1%	-0.24 [-0.42, -0.06]
Qi-Hang Su 2024	4.47	1.22	30	5.13	1.2	30	7.8%	-0.66 [-1.27, -0.05]
Sen Li 2021	2.51	0.37	30	3.02	0.25	30	10.2%	-0.51 [-0.67, -0.35]
Shaohui Shi 2017 a	4.3	3.2	159	5.8	3.2	127	7.0%	-1.50 [-2.25, -0.75]
Tao Zeng 2018	2.4	0.49	30	3.66	0.6	30	9.8%	-1.26 [-1.54, -0.98]
Xinyu Chen 2015 a	0.54	0.13	63	1.22	0.27	63	10.4%	-0.68 [-0.75, -0.61]
Zhenlong Bai 2015 a	2.29	1.89	17	3.29	2.34	17	3.7%	-1.00 [-2.43, 0.43]
Zhi Li 2021	3.51	0.56	100	4.19	0.54	100	10.2%	-0.68 [-0.83, -0.53]
**Total (95% CI)**			**651**			**619**	**100.0%**	**-1.05 [-1.39, -0.70]**

Heterogeneity: Tau² = 0.30; Chi² = 596.31, df = 10 (P < 0.00001); I² = 98%
Test for overall effect: Z = 5.97 (P < 0.00001)

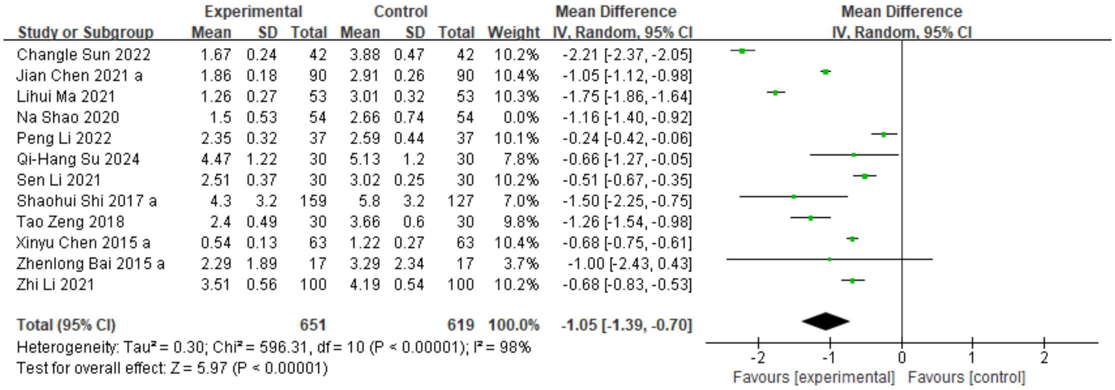

(d) Excluding Study D

Study or Subgroup	Experimental Mean	Experimental SD	Experimental Total	Control Mean	Control SD	Control Total	Weight	Mean Difference IV, Random, 95% CI
Changle Sun 2022	1.67	0.24	42	3.88	0.47	42	10.3%	-2.21 [-2.37, -2.05]
Jian Chen 2021 a	1.86	0.18	90	2.91	0.26	90	10.5%	-1.05 [-1.12, -0.98]
Lihui Ma 2021	1.26	0.27	53	3.01	0.32	53	10.4%	-1.75 [-1.86, -1.64]
Na Shao 2020	1.5	0.53	54	2.66	0.74	54	10.0%	-1.16 [-1.40, -0.92]
Peng Li 2022	2.35	0.32	37	2.59	0.44	37	0.0%	-0.24 [-0.42, -0.06]
Qi-Hang Su 2024	4.47	1.22	30	5.13	1.2	30	7.7%	-0.66 [-1.27, -0.05]
Sen Li 2021	2.51	0.37	30	3.02	0.25	30	10.3%	-0.51 [-0.67, -0.35]
Shaohui Shi 2017 a	4.3	3.2	159	5.8	3.2	127	6.8%	-1.50 [-2.25, -0.75]
Tao Zeng 2018	2.4	0.49	30	3.66	0.6	30	9.8%	-1.26 [-1.54, -0.98]
Xinyu Chen 2015 a	0.54	0.13	63	1.22	0.27	63	10.5%	-0.68 [-0.75, -0.61]
Zhenlong Bai 2015 a	2.29	1.89	17	3.29	2.34	17	3.5%	-1.00 [-2.43, 0.43]
Zhi Li 2021	3.51	0.56	100	4.19	0.54	100	10.3%	-0.68 [-0.83, -0.53]
**Total (95% CI)**			**668**			**636**	**100.0%**	**-1.14 [-1.47, -0.81]**

Heterogeneity: Tau² = 0.27; Chi² = 519.56, df = 10 (P < 0.00001); I² = 98%
Test for overall effect: Z = 6.81 (P < 0.00001)

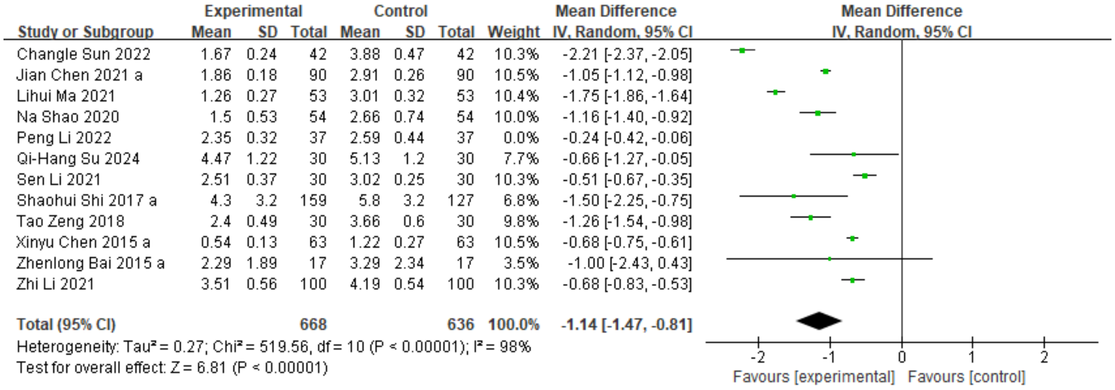

(e) Excluding Study E

Study or Subgroup	Experimental Mean	Experimental SD	Experimental Total	Control Mean	Control SD	Control Total	Weight	Mean Difference IV, Random, 95% CI
Changle Sun 2022	1.67	0.24	42	3.88	0.47	42	10.0%	-2.21 [-2.37, -2.05]
Jian Chen 2021 a	1.86	0.18	90	2.91	0.26	90	10.2%	-1.05 [-1.12, -0.98]
Lihui Ma 2021	1.26	0.27	53	3.01	0.32	53	10.1%	-1.75 [-1.86, -1.64]
Na Shao 2020	1.5	0.53	54	2.66	0.74	54	9.7%	-1.16 [-1.40, -0.92]
Peng Li 2022	2.35	0.32	37	2.59	0.44	37	9.9%	-0.24 [-0.42, -0.06]
Qi-Hang Su 2024	4.47	1.22	30	5.13	1.2	30	0.0%	-0.66 [-1.27, -0.05]
Sen Li 2021	2.51	0.37	30	3.02	0.25	30	10.0%	-0.51 [-0.67, -0.35]
Shaohui Shi 2017 a	4.3	3.2	159	5.8	3.2	127	6.8%	-1.50 [-2.25, -0.75]
Tao Zeng 2018	2.4	0.49	30	3.66	0.6	30	9.6%	-1.26 [-1.54, -0.98]
Xinyu Chen 2015 a	0.54	0.13	63	1.22	0.27	63	10.2%	-0.68 [-0.75, -0.61]
Zhenlong Bai 2015 a	2.29	1.89	17	3.29	2.34	17	3.6%	-1.00 [-2.43, 0.43]
Zhi Li 2021	3.51	0.56	100	4.19	0.54	100	10.0%	-0.68 [-0.83, -0.53]
**Total (95% CI)**			**675**			**643**	**100.0%**	**-1.09 [-1.42, -0.75]**

Heterogeneity: Tau² = 0.29; Chi² = 596.50, df = 10 (P < 0.00001); I² = 98%
Test for overall effect: Z = 6.36 (P < 0.00001)

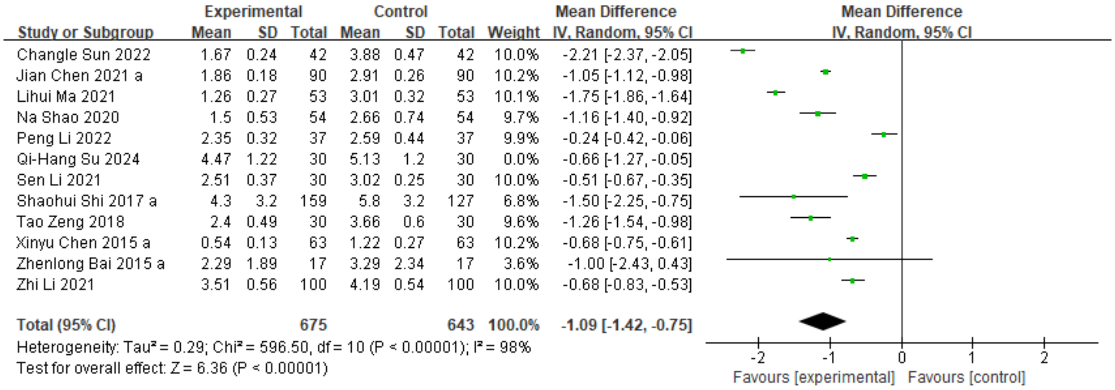

(f) Excluding Study F

Study or Subgroup	Experimental Mean	Experimental SD	Experimental Total	Control Mean	Control SD	Control Total	Weight	Mean Difference IV, Random, 95% CI
Changle Sun 2022	1.67	0.24	42	3.88	0.47	42	10.2%	-2.21 [-2.37, -2.05]
Jian Chen 2021 a	1.86	0.18	90	2.91	0.26	90	10.4%	-1.05 [-1.12, -0.98]
Lihui Ma 2021	1.26	0.27	53	3.01	0.32	53	10.3%	-1.75 [-1.86, -1.64]
Na Shao 2020	1.5	0.53	54	2.66	0.74	54	9.9%	-1.16 [-1.40, -0.92]
Peng Li 2022	2.35	0.32	37	2.59	0.44	37	10.2%	-0.24 [-0.42, -0.06]
Qi-Hang Su 2024	4.47	1.22	30	5.13	1.2	30	7.8%	-0.66 [-1.27, -0.05]
Sen Li 2021	2.51	0.37	30	3.02	0.25	30	0.0%	-0.51 [-0.67, -0.35]
Shaohui Shi 2017 a	4.3	3.2	159	5.8	3.2	127	7.0%	-1.50 [-2.25, -0.75]
Tao Zeng 2018	2.4	0.49	30	3.66	0.6	30	9.8%	-1.26 [-1.54, -0.98]
Xinyu Chen 2015 a	0.54	0.13	63	1.22	0.27	63	10.4%	-0.68 [-0.75, -0.61]
Zhenlong Bai 2015 a	2.29	1.89	17	3.29	2.34	17	3.7%	-1.00 [-2.43, 0.43]
Zhi Li 2021	3.51	0.56	100	4.19	0.54	100	10.2%	-0.68 [-0.83, -0.53]
**Total (95% CI)**			**675**			**643**	**100.0%**	**-1.11 [-1.45, -0.77]**

Heterogeneity: Tau² = 0.29; Chi² = 557.56, df = 10 (P < 0.00001); I² = 98%
Test for overall effect: Z = 6.39 (P < 0.00001)

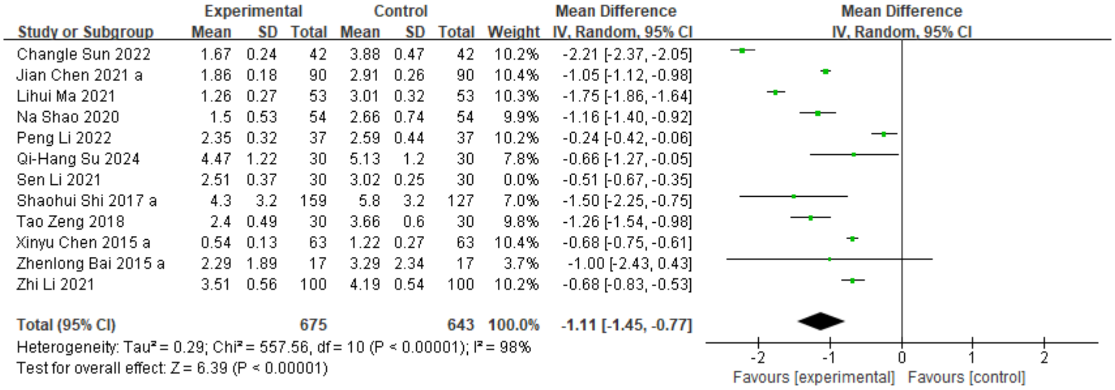

(g) Excluding Study G

Study or Subgroup	Experimental Mean	Experimental SD	Experimental Total	Control Mean	Control SD	Control Total	Weight	Mean Difference IV, Random, 95% CI
Changle Sun 2022	1.67	0.24	42	3.88	0.47	42	9.9%	-2.21 [-2.37, -2.05]
Jian Chen 2021 a	1.86	0.18	90	2.91	0.26	90	10.1%	-1.05 [-1.12, -0.98]
Lihui Ma 2021	1.26	0.27	53	3.01	0.32	53	10.0%	-1.75 [-1.86, -1.64]
Na Shao 2020	1.5	0.53	54	2.66	0.74	54	9.6%	-1.16 [-1.40, -0.92]
Peng Li 2022	2.35	0.32	37	2.59	0.44	37	9.9%	-0.24 [-0.42, -0.06]
Qi-Hang Su 2024	4.47	1.22	30	5.13	1.2	30	7.6%	-0.66 [-1.27, -0.05]
Sen Li 2021	2.51	0.37	30	3.02	0.25	30	9.9%	-0.51 [-0.67, -0.35]
Shaohui Shi 2017 a	4.3	3.2	159	5.8	3.2	127	0.0%	-1.50 [-2.25, -0.75]
Tao Zeng 2018	2.4	0.49	30	3.66	0.6	30	9.5%	-1.26 [-1.54, -0.98]
Xinyu Chen 2015 a	0.54	0.13	63	1.22	0.27	63	10.1%	-0.68 [-0.75, -0.61]
Zhenlong Bai 2015 a	2.29	1.89	17	3.29	2.34	17	3.5%	-1.00 [-2.43, 0.43]
Zhi Li 2021	3.51	0.56	100	4.19	0.54	100	9.9%	-0.68 [-0.83, -0.53]
**Total (95% CI)**			**546**			**546**	**100.0%**	**-1.03 [-1.36, -0.69]**

Heterogeneity: Tau² = 0.29; Chi² = 596.13, df = 10 (P < 0.00001); I² = 98%
Test for overall effect: Z = 6.04 (P < 0.00001)

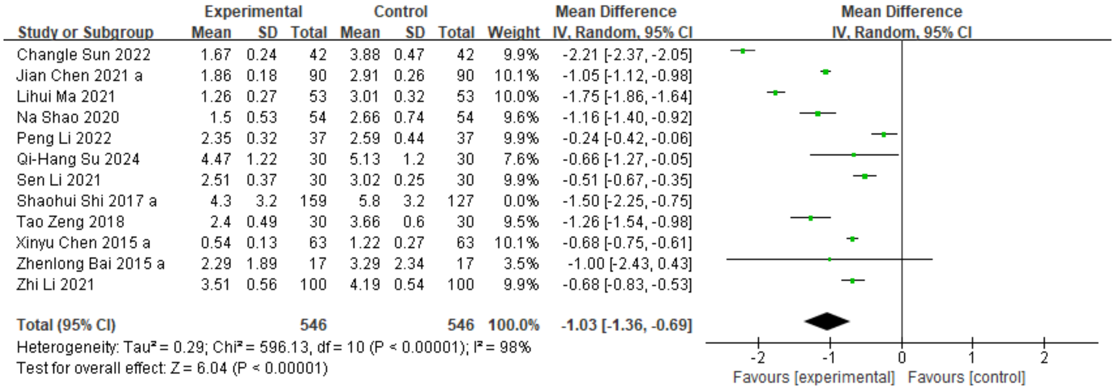

(h) Excluding Study H

Study or Subgroup	Experimental Mean	Experimental SD	Experimental Total	Control Mean	Control SD	Control Total	Weight	Mean Difference IV, Random, 95% CI
Changle Sun 2022	1.67	0.24	42	3.88	0.47	42	10.2%	-2.21 [-2.37, -2.05]
Jian Chen 2021 a	1.86	0.18	90	2.91	0.26	90	10.4%	-1.05 [-1.12, -0.98]
Lihui Ma 2021	1.26	0.27	53	3.01	0.32	53	10.3%	-1.75 [-1.86, -1.64]
Na Shao 2020	1.5	0.53	54	2.66	0.74	54	9.9%	-1.16 [-1.40, -0.92]
Peng Li 2022	2.35	0.32	37	2.59	0.44	37	10.1%	-0.24 [-0.42, -0.06]
Qi-Hang Su 2024	4.47	1.22	30	5.13	1.2	30	7.8%	-0.66 [-1.27, -0.05]
Sen Li 2021	2.51	0.37	30	3.02	0.25	30	10.2%	-0.51 [-0.67, -0.35]
Shaohui Shi 2017 a	4.3	3.2	159	5.8	3.2	127	7.0%	-1.50 [-2.25, -0.75]
Tao Zeng 2018	2.4	0.49	30	3.66	0.6	30	0.0%	-1.26 [-1.54, -0.98]
Xinyu Chen 2015 a	0.54	0.13	63	1.22	0.27	63	10.4%	-0.68 [-0.75, -0.61]
Zhenlong Bai 2015 a	2.29	1.89	17	3.29	2.34	17	3.7%	-1.00 [-2.43, 0.43]
Zhi Li 2021	3.51	0.56	100	4.19	0.54	100	10.2%	-0.68 [-0.83, -0.53]
**Total (95% CI)**			**675**			**643**	**100.0%**	**-1.04 [-1.38, -0.70]**

Heterogeneity: Tau² = 0.29; Chi² = 594.64, df = 10 (P < 0.00001); I² = 98%
Test for overall effect: Z = 5.95 (P < 0.00001)

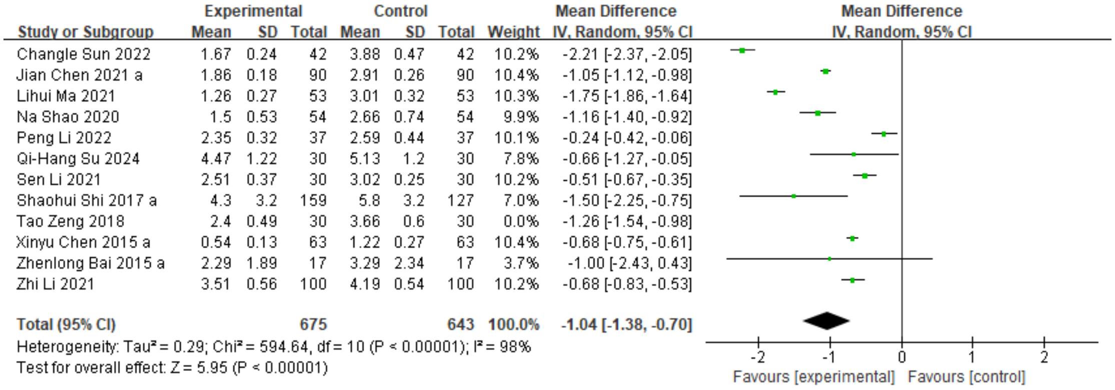

(i) Excluding Study I

Study or Subgroup	Experimental Mean	Experimental SD	Experimental Total	Control Mean	Control SD	Control Total	Weight	Mean Difference IV, Random, 95% CI
Changle Sun 2022	1.67	0.24	42	3.88	0.47	42	10.1%	-2.21 [-2.37, -2.05]
Jian Chen 2021 a	1.86	0.18	90	2.91	0.26	90	10.3%	-1.05 [-1.12, -0.98]
Lihui Ma 2021	1.26	0.27	53	3.01	0.32	53	10.2%	-1.75 [-1.86, -1.64]
Na Shao 2020	1.5	0.53	54	2.66	0.74	54	9.9%	-1.16 [-1.40, -0.92]
Peng Li 2022	2.35	0.32	37	2.59	0.44	37	10.1%	-0.24 [-0.42, -0.06]
Qi-Hang Su 2024	4.47	1.22	30	5.13	1.2	30	8.0%	-0.66 [-1.27, -0.05]
Sen Li 2021	2.51	0.37	30	3.02	0.25	30	10.1%	-0.51 [-0.67, -0.35]
Shaohui Shi 2017 a	4.3	3.2	159	5.8	3.2	127	7.3%	-1.50 [-2.25, -0.75]
Tao Zeng 2018	2.4	0.49	30	3.66	0.6	30	9.8%	-1.26 [-1.54, -0.98]
Xinyu Chen 2015 a	0.54	0.13	63	1.22	0.27	63	0.0%	-0.68 [-0.75, -0.61]
Zhenlong Bai 2015 a	2.29	1.89	17	3.29	2.34	17	4.0%	-1.00 [-2.43, 0.43]
Zhi Li 2021	3.51	0.56	100	4.19	0.54	100	10.1%	-0.68 [-0.83, -0.53]
**Total (95% CI)**			**642**			**610**	**100.0%**	**-1.10 [-1.46, -0.73]**

Heterogeneity: Tau² = 0.34; Chi² = 492.31, df = 10 (P < 0.00001); I² = 98%
Test for overall effect: Z = 5.83 (P < 0.00001)

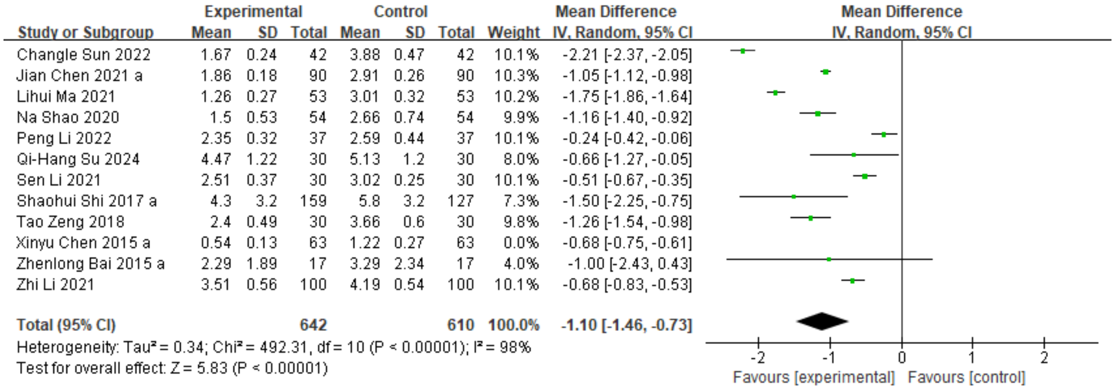

(j) Excluding Study J

Study or Subgroup	Experimental Mean	Experimental SD	Experimental Total	Control Mean	Control SD	Control Total	Weight	Mean Difference IV, Random, 95% CI
Changle Sun 2022	1.67	0.24	42	3.88	0.47	42	9.6%	-2.21 [-2.37, -2.05]
Jian Chen 2021 a	1.86	0.18	90	2.91	0.26	90	9.8%	-1.05 [-1.12, -0.98]
Lihui Ma 2021	1.26	0.27	53	3.01	0.32	53	9.7%	-1.75 [-1.86, -1.64]
Na Shao 2020	1.5	0.53	54	2.66	0.74	54	9.3%	-1.16 [-1.40, -0.92]
Peng Li 2022	2.35	0.32	37	2.59	0.44	37	9.6%	-0.24 [-0.42, -0.06]
Qi-Hang Su 2024	4.47	1.22	30	5.13	1.2	30	7.3%	-0.66 [-1.27, -0.05]
Sen Li 2021	2.51	0.37	30	3.02	0.25	30	9.6%	-0.51 [-0.67, -0.35]
Shaohui Shi 2017 a	4.3	3.2	159	5.8	3.2	127	6.5%	-1.50 [-2.25, -0.75]
Tao Zeng 2018	2.4	0.49	30	3.66	0.6	30	9.2%	-1.26 [-1.54, -0.98]
Xinyu Chen 2015 a	0.54	0.13	63	1.22	0.27	63	9.8%	-0.68 [-0.75, -0.61]
Zhenlong Bai 2015 a	2.29	1.89	17	3.29	2.34	17	0.0%	-1.00 [-2.43, 0.43]
Zhi Li 2021	3.51	0.56	100	4.19	0.54	100	9.6%	-0.68 [-0.83, -0.53]
**Total (95% CI)**			**688**			**656**	**100.0%**	**-1.06 [-1.39, -0.73]**

Heterogeneity: Tau² = 0.29; Chi² = 597.77, df = 10 (P < 0.00001); I² = 98%
Test for overall effect: Z = 6.32 (P < 0.00001)

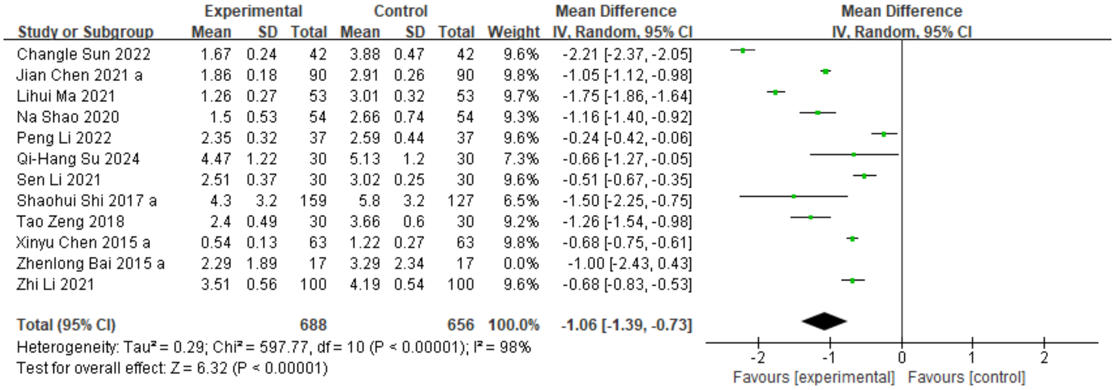

(k) Excluding Study K

Study or Subgroup	Experimental Mean	Experimental SD	Experimental Total	Control Mean	Control SD	Control Total	Weight	Mean Difference IV, Random, 95% CI
Changle Sun 2022	1.67	0.24	42	3.88	0.47	42	10.2%	-2.21 [-2.37, -2.05]
Jian Chen 2021 a	1.86	0.18	90	2.91	0.26	90	10.4%	-1.05 [-1.12, -0.98]
Lihui Ma 2021	1.26	0.27	53	3.01	0.32	53	10.3%	-1.75 [-1.86, -1.64]
Na Shao 2020	1.5	0.53	54	2.66	0.74	54	9.9%	-1.16 [-1.40, -0.92]
Peng Li 2022	2.35	0.32	37	2.59	0.44	37	10.1%	-0.24 [-0.42, -0.06]
Qi-Hang Su 2024	4.47	1.22	30	5.13	1.2	30	7.9%	-0.66 [-1.27, -0.05]
Sen Li 2021	2.51	0.37	30	3.02	0.25	30	10.2%	-0.51 [-0.67, -0.35]
Shaohui Shi 2017 a	4.3	3.2	159	5.8	3.2	127	7.1%	-1.50 [-2.25, -0.75]
Tao Zeng 2018	2.4	0.49	30	3.66	0.6	30	9.8%	-1.26 [-1.54, -0.98]
Xinyu Chen 2015 a	0.54	0.13	63	1.22	0.27	63	10.4%	-0.68 [-0.75, -0.61]
Zhenlong Bai 2015 a	2.29	1.89	17	3.29	2.34	17	3.8%	-1.00 [-2.43, 0.43]
Zhi Li 2021	3.51	0.56	100	4.19	0.54	100	0.0%	-0.68 [-0.83, -0.53]
**Total (95% CI)**			**605**			**573**	**100.0%**	**-1.10 [-1.44, -0.75]**

Heterogeneity: Tau² = 0.30; Chi² = 578.34, df = 10 (P < 0.00001); I² = 98%
Test for overall effect: Z = 6.16 (P < 0.00001)

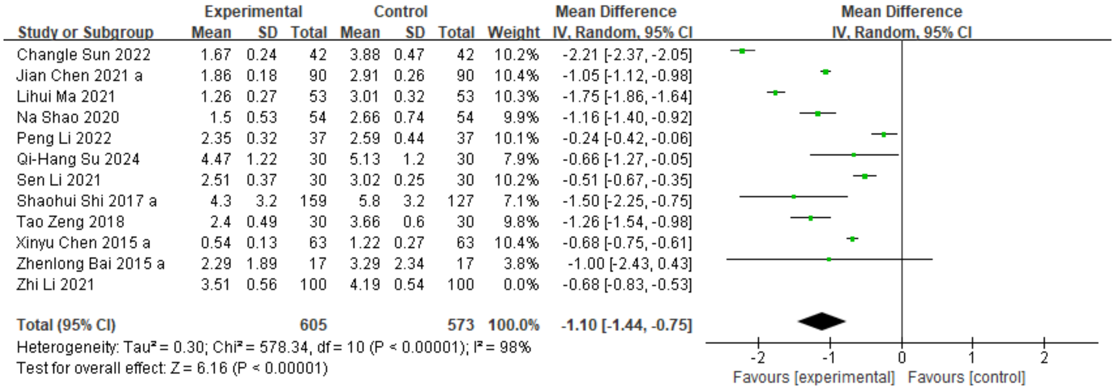

(l) Excluding Study L
