# Supplementary material for: Effect of Medical Chitosan on Clinical Efficacy and Pain in Knee Osteoarthritis: A Systematic Review and Meta-Analysis
Source: Diseases. 2026 Jul 14;14(7):252. doi: 10.3390/diseases14070252 (PMC13408866; doi:10.3390/diseases14070252)
Supplement: Supplementary file 1 [file diseases-14-00252-s001.zip › Supplementary_Figure_S2_Leave_one_out sensitivity analysis forest plots for clinical efficacy.pdf]

Supplementary Figure S2. Leave-one-out sensitivity analysis forest plots for clinical efficacy.

Study or Subgroup	Experimental Events	Experimental Total	Control Events	Control Total	Weight	Odds Ratio M-H, Fixed, 95% CI
Changle Sun 2022	39	42	32	42	0.0%	4.06 [1.03, 16.02]
Jian Chen 2021 a	90	90	83	90	3.6%	16.26 [0.91, 289.07]
Lihui Ma 2021	49	53	43	53	25.7%	2.85 [0.83, 9.74]
Na Shao 2020	51	54	43	54	18.9%	4.35 [1.14, 16.60]
Peng Li 2022	35	37	28	37	12.0%	5.63 [1.12, 28.16]
Sen Li 2021	28	30	25	30	13.2%	2.80 [0.50, 15.73]
Tao Zeng 2018	30	30	28	30	3.6%	5.35 [0.25, 116.31]
Xinyu Chen 2015 a	61	63	53	63	13.3%	5.75 [1.21, 27.44]
Yanqiong Jiang 2024	26	27	21	27	6.2%	7.43 [0.83, 66.62]
Zhi Li 2021	100	100	87	100	3.4%	31.01 [1.82, 529.29]
**Total (95% CI)**		**484**		**484**	**100.0%**	**5.67 [3.21, 10.03]**
Total events	470		411			

Heterogeneity: Chi² = 3.95, df = 8 (P = 0.86); I² = 0%
Test for overall effect: Z = 5.97 (P < 0.00001)

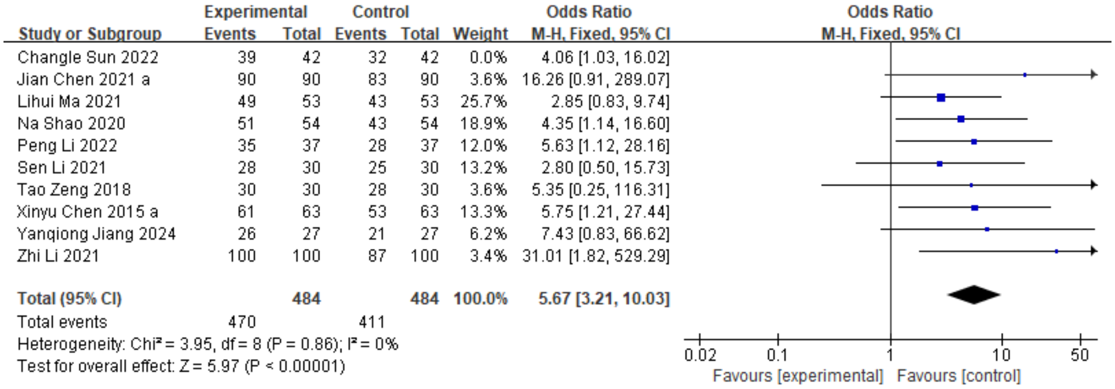

(a) Excluding Study A

Study or Subgroup	Experimental Events	Experimental Total	Control Events	Control Total	Weight	Odds Ratio M-H, Fixed, 95% CI
Changle Sun 2022	39	42	32	42	15.8%	4.06 [1.03, 16.02]
Jian Chen 2021 a	90	90	83	90	0.0%	16.26 [0.91, 289.07]
Lihui Ma 2021	49	53	43	53	22.5%	2.85 [0.83, 9.74]
Na Shao 2020	51	54	43	54	16.5%	4.35 [1.14, 16.60]
Peng Li 2022	35	37	28	37	10.5%	5.63 [1.12, 28.16]
Sen Li 2021	28	30	25	30	11.5%	2.80 [0.50, 15.73]
Tao Zeng 2018	30	30	28	30	3.2%	5.35 [0.25, 116.31]
Xinyu Chen 2015 a	61	63	53	63	11.6%	5.75 [1.21, 27.44]
Yanqiong Jiang 2024	26	27	21	27	5.4%	7.43 [0.83, 66.62]
Zhi Li 2021	100	100	87	100	3.0%	31.01 [1.82, 529.29]
**Total (95% CI)**		**436**		**436**	**100.0%**	**5.08 [2.97, 8.69]**
Total events	419		360			

Heterogeneity: Chi² = 3.18, df = 8 (P = 0.92); I² = 0%
Test for overall effect: Z = 5.94 (P < 0.00001)

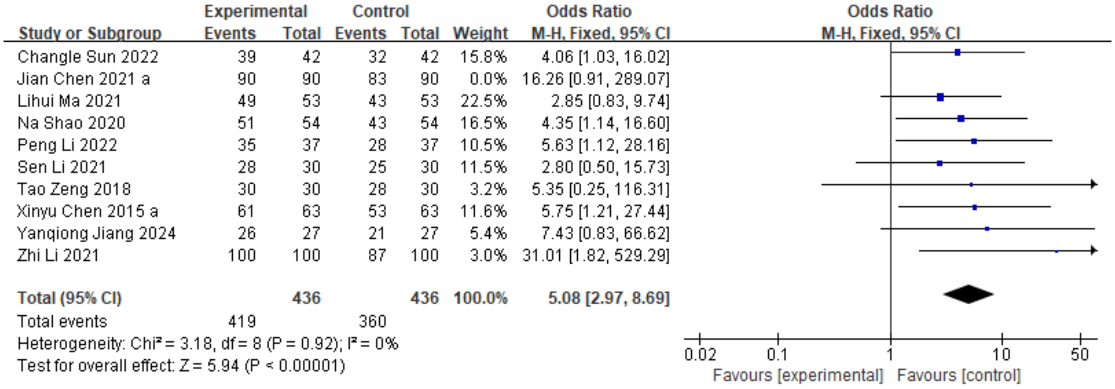

(b) Excluding Study B

Study or Subgroup	Experimental Events	Experimental Total	Control Events	Control Total	Weight	Odds Ratio M-H, Fixed, 95% CI
Changle Sun 2022	39	42	32	42	19.6%	4.06 [1.03, 16.02]
Jian Chen 2021 a	90	90	83	90	3.9%	16.26 [0.91, 289.07]
Lihui Ma 2021	49	53	43	53	0.0%	2.85 [0.83, 9.74]
Na Shao 2020	51	54	43	54	20.5%	4.35 [1.14, 16.60]
Peng Li 2022	35	37	28	37	13.0%	5.63 [1.12, 28.16]
Sen Li 2021	28	30	25	30	14.3%	2.80 [0.50, 15.73]
Tao Zeng 2018	30	30	28	30	3.9%	5.35 [0.25, 116.31]
Xinyu Chen 2015 a	61	63	53	63	14.4%	5.75 [1.21, 27.44]
Yanqiong Jiang 2024	26	27	21	27	6.7%	7.43 [0.83, 66.62]
Zhi Li 2021	100	100	87	100	3.7%	31.01 [1.82, 529.29]
**Total (95% CI)**		**473**		**473**	**100.0%**	**6.14 [3.42, 11.04]**
Total events	460		400			

Heterogeneity: Chi² = 3.14, df = 8 (P = 0.92); I² = 0%
Test for overall effect: Z = 6.07 (P < 0.00001)

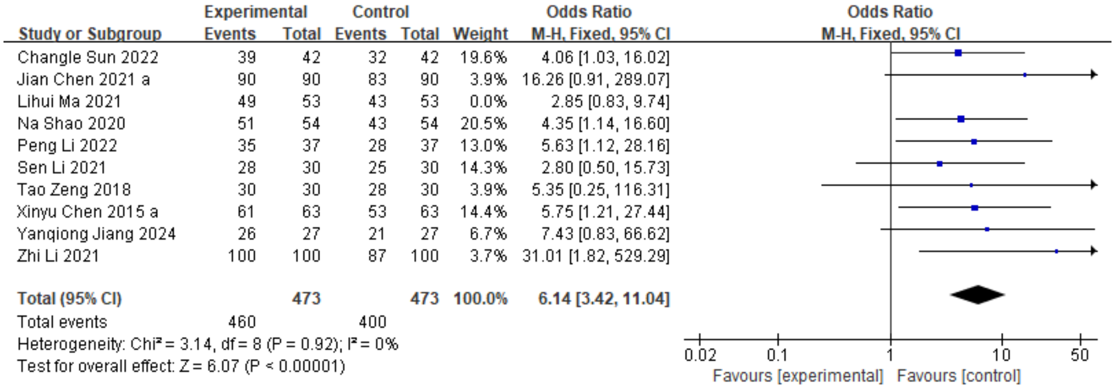

(c) Excluding Study C

Study or Subgroup	Experimental Events	Experimental Total	Control Events	Control Total	Weight	Odds Ratio M-H, Fixed, 95% CI
Changle Sun 2022	39	42	32	42	18.3%	4.06 [1.03, 16.02]
Jian Chen 2021 a	90	90	83	90	3.7%	16.26 [0.91, 289.07]
Lihui Ma 2021	49	53	43	53	25.9%	2.85 [0.83, 9.74]
Na Shao 2020	51	54	43	54	0.0%	4.35 [1.14, 16.60]
Peng Li 2022	35	37	28	37	12.1%	5.63 [1.12, 28.16]
Sen Li 2021	28	30	25	30	13.3%	2.80 [0.50, 15.73]
Tao Zeng 2018	30	30	28	30	3.7%	5.35 [0.25, 116.31]
Xinyu Chen 2015 a	61	63	53	63	13.4%	5.75 [1.21, 27.44]
Yanqiong Jiang 2024	26	27	21	27	6.2%	7.43 [0.83, 66.62]
Zhi Li 2021	100	100	87	100	3.5%	31.01 [1.82, 529.29]
**Total (95% CI)**		**472**		**472**	**100.0%**	**5.63 [3.18, 9.98]**
Total events	458		400			

Heterogeneity: Chi² = 4.00, df = 8 (P = 0.86); I² = 0%
Test for overall effect: Z = 5.92 (P < 0.00001)

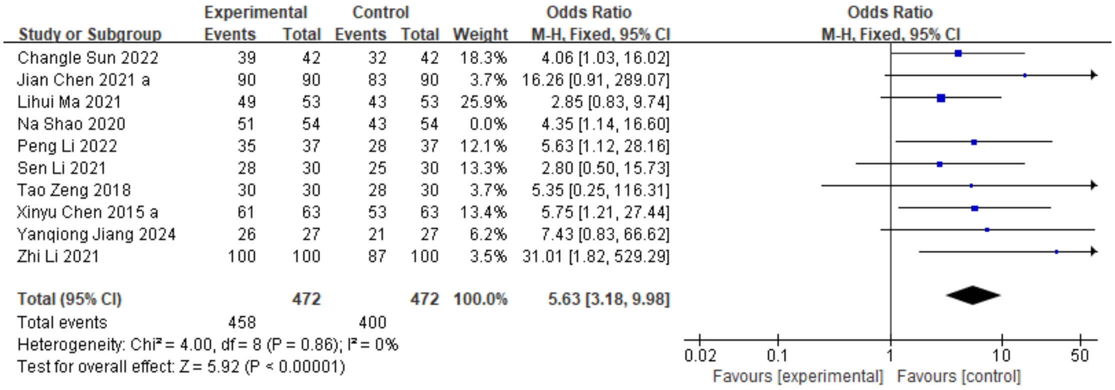

(d) Excluding Study D

Study or Subgroup	Experimental Events	Experimental Total	Control Events	Control Total	Weight	Odds Ratio M-H, Fixed, 95% CI
Changle Sun 2022	39	42	32	42	17.1%	4.06 [1.03, 16.02]
Jian Chen 2021 a	90	90	83	90	3.4%	16.26 [0.91, 289.07]
Lihui Ma 2021	49	53	43	53	24.2%	2.85 [0.83, 9.74]
Na Shao 2020	51	54	43	54	17.8%	4.35 [1.14, 16.60]
Peng Li 2022	35	37	28	37	0.0%	5.63 [1.12, 28.16]
Sen Li 2021	28	30	25	30	12.4%	2.80 [0.50, 15.73]
Tao Zeng 2018	30	30	28	30	3.4%	5.35 [0.25, 116.31]
Xinyu Chen 2015 a	61	63	53	63	12.6%	5.75 [1.21, 27.44]
Yanqiong Jiang 2024	26	27	21	27	5.8%	7.43 [0.83, 66.62]
Zhi Li 2021	100	100	87	100	3.2%	31.01 [1.82, 529.29]
**Total (95% CI)**		**489**		**489**	**100.0%**	**5.40 [3.10, 9.42]**
Total events	474		415			

Heterogeneity: Chi² = 3.97, df = 8 (P = 0.86); I² = 0%
Test for overall effect: Z = 5.94 (P < 0.00001)

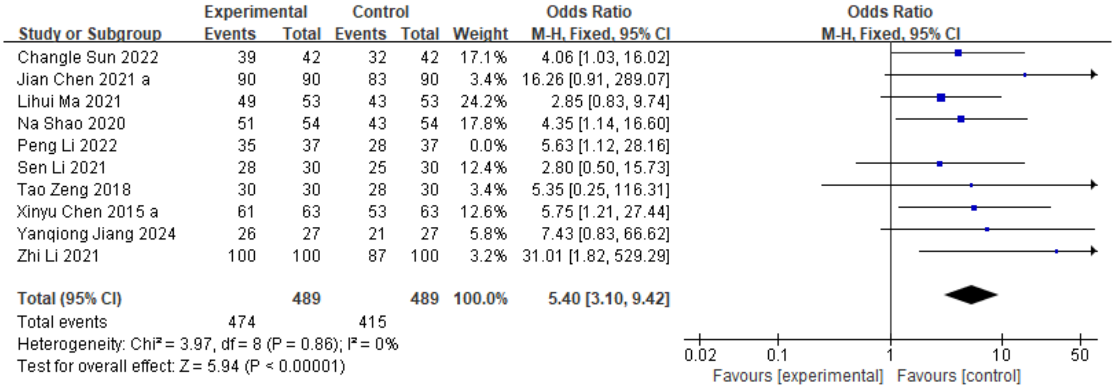

(e) Excluding Study E

Study or Subgroup	Experimental Events	Experimental Total	Control Events	Control Total	Weight	Odds Ratio M-H, Fixed, 95% CI
Changle Sun 2022	39	42	32	42	17.3%	4.06 [1.03, 16.02]
Jian Chen 2021 a	90	90	83	90	3.5%	16.26 [0.91, 289.07]
Lihui Ma 2021	49	53	43	53	24.5%	2.85 [0.83, 9.74]
Na Shao 2020	51	54	43	54	18.0%	4.35 [1.14, 16.60]
Peng Li 2022	35	37	28	37	11.4%	5.63 [1.12, 28.16]
Sen Li 2021	28	30	25	30	0.0%	2.80 [0.50, 15.73]
Tao Zeng 2018	30	30	28	30	3.5%	5.35 [0.25, 116.31]
Xinyu Chen 2015 a	61	63	53	63	12.7%	5.75 [1.21, 27.44]
Yanqiong Jiang 2024	26	27	21	27	5.9%	7.43 [0.83, 66.62]
Zhi Li 2021	100	100	87	100	3.3%	31.01 [1.82, 529.29]
**Total (95% CI)**		**496**		**496**	**100.0%**	**5.76 [3.31, 10.02]**
Total events	481		418			

Heterogeneity: Chi² = 3.58, df = 8 (P = 0.89); I² = 0%
Test for overall effect: Z = 6.19 (P < 0.00001)

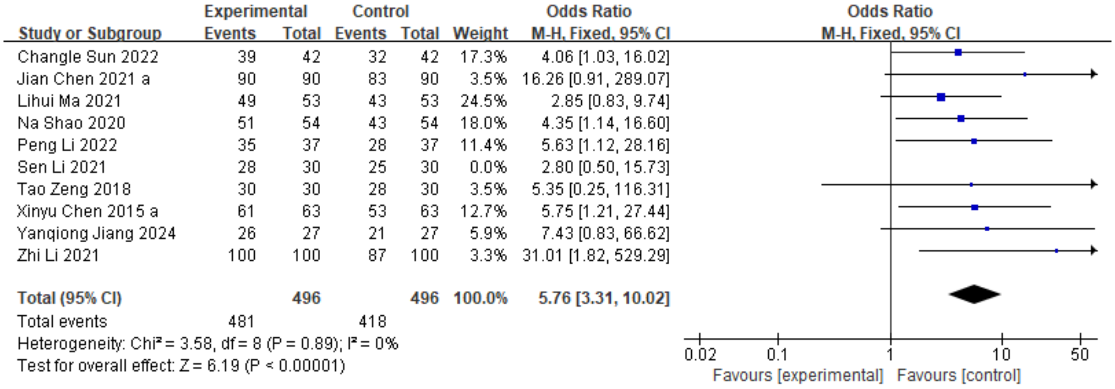

(f) Excluding Study F

Study or Subgroup	Experimental Events	Experimental Total	Control Events	Control Total	Weight	Odds Ratio M-H, Fixed, 95% CI
Changle Sun 2022	39	42	32	42	15.8%	4.06 [1.03, 16.02]
Jian Chen 2021 a	90	90	83	90	3.2%	16.26 [0.91, 289.07]
Lihui Ma 2021	49	53	43	53	22.5%	2.85 [0.83, 9.74]
Na Shao 2020	51	54	43	54	16.5%	4.35 [1.14, 16.60]
Peng Li 2022	35	37	28	37	10.5%	5.63 [1.12, 28.16]
Sen Li 2021	28	30	25	30	11.5%	2.80 [0.50, 15.73]
Tao Zeng 2018	30	30	28	30	0.0%	5.35 [0.25, 116.31]
Xinyu Chen 2015 a	61	63	53	63	11.6%	5.75 [1.21, 27.44]
Yanqiong Jiang 2024	26	27	21	27	5.4%	7.43 [0.83, 66.62]
Zhi Li 2021	100	100	87	100	3.0%	31.01 [1.82, 529.29]
**Total (95% CI)**		**496**		**496**	**100.0%**	**5.43 [3.18, 9.26]**
Total events	479		415			

Heterogeneity: Chi² = 3.99, df = 8 (P = 0.86); I² = 0%
Test for overall effect: Z = 6.21 (P < 0.00001)

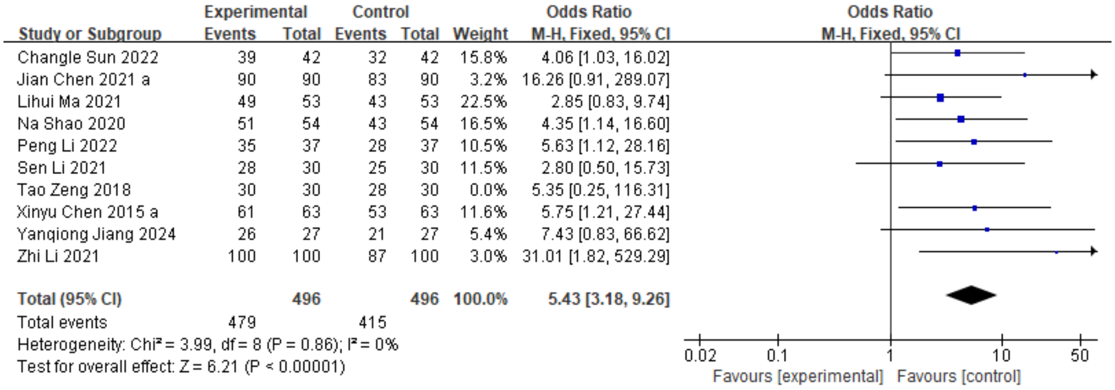

(g) Excluding Study G

Study or Subgroup	Experimental Events	Experimental Total	Control Events	Control Total	Weight	Odds Ratio M-H, Fixed, 95% CI
Changle Sun 2022	39	42	32	42	17.3%	4.06 [1.03, 16.02]
Jian Chen 2021 a	90	90	83	90	3.5%	16.26 [0.91, 289.07]
Lihui Ma 2021	49	53	43	53	24.5%	2.85 [0.83, 9.74]
Na Shao 2020	51	54	43	54	18.1%	4.35 [1.14, 16.60]
Peng Li 2022	35	37	28	37	11.4%	5.63 [1.12, 28.16]
Sen Li 2021	28	30	25	30	12.6%	2.80 [0.50, 15.73]
Tao Zeng 2018	30	30	28	30	3.5%	5.35 [0.25, 116.31]
Xinyu Chen 2015 a	61	63	53	63	0.0%	5.75 [1.21, 27.44]
Yanqiong Jiang 2024	26	27	21	27	5.9%	7.43 [0.83, 66.62]
Zhi Li 2021	100	100	87	100	3.3%	31.01 [1.82, 529.29]
**Total (95% CI)**		**463**		**463**	**100.0%**	**5.38 [3.08, 9.41]**
Total events	448		390			

Heterogeneity: Chi² = 3.95, df = 8 (P = 0.86); I² = 0%
Test for overall effect: Z = 5.91 (P < 0.00001)

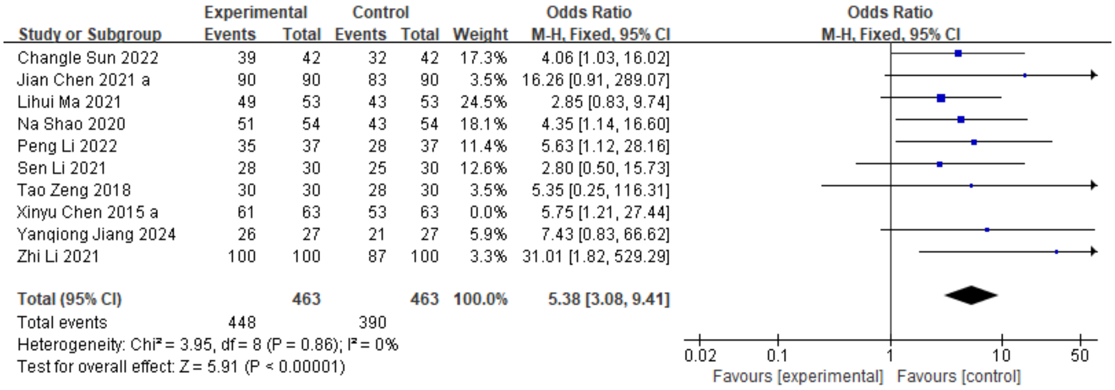

(h) Excluding Study H

Study or Subgroup	Experimental Events	Experimental Total	Control Events	Control Total	Weight	Odds Ratio M-H, Fixed, 95% CI
Changle Sun 2022	39	42	32	42	15.8%	4.06 [1.03, 16.02]
Jian Chen 2021 a	90	90	83	90	3.2%	16.26 [0.91, 289.07]
Lihui Ma 2021	49	53	43	53	22.4%	2.85 [0.83, 9.74]
Na Shao 2020	51	54	43	54	16.5%	4.35 [1.14, 16.60]
Peng Li 2022	35	37	28	37	10.5%	5.63 [1.12, 28.16]
Sen Li 2021	28	30	25	30	11.5%	2.80 [0.50, 15.73]
Tao Zeng 2018	30	30	28	30	3.2%	5.35 [0.25, 116.31]
Xinyu Chen 2015 a	61	63	53	63	11.6%	5.75 [1.21, 27.44]
Yanqiong Jiang 2024	26	27	21	27	5.4%	7.43 [0.83, 66.62]
Zhi Li 2021	100	100	87	100	0.0%	31.01 [1.82, 529.29]
**Total (95% CI)**		**426**		**426**	**100.0%**	**4.66 [2.71, 8.01]**
Total events	409		356			

Heterogeneity: Chi² = 2.03, df = 8 (P = 0.98); I² = 0%
Test for overall effect: Z = 5.57 (P < 0.00001)

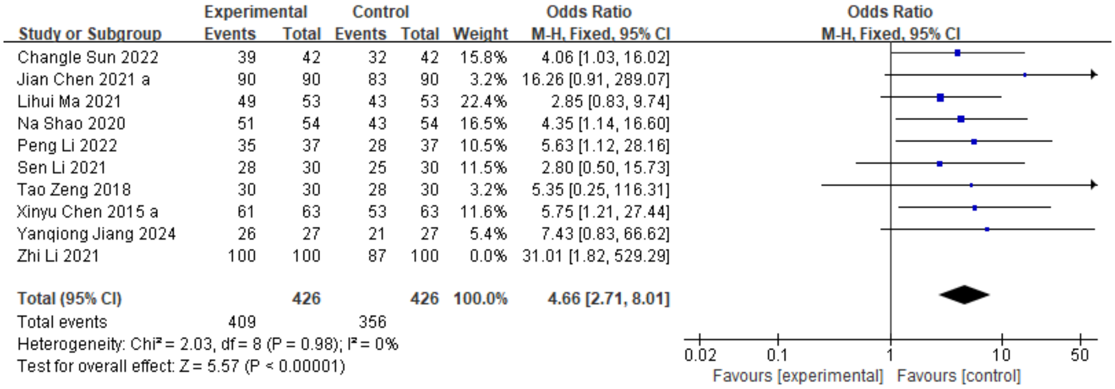

(i) Excluding Study I

Study or Subgroup	Experimental Events	Experimental Total	Control Events	Control Total	Weight	Odds Ratio M-H, Fixed, 95% CI
Changle Sun 2022	39	42	32	42	16.2%	4.06 [1.03, 16.02]
Jian Chen 2021 a	90	90	83	90	3.2%	16.26 [0.91, 289.07]
Lihui Ma 2021	49	53	43	53	23.0%	2.85 [0.83, 9.74]
Na Shao 2020	51	54	43	54	16.9%	4.35 [1.14, 16.60]
Peng Li 2022	35	37	28	37	10.7%	5.63 [1.12, 28.16]
Sen Li 2021	28	30	25	30	11.8%	2.80 [0.50, 15.73]
Tao Zeng 2018	30	30	28	30	3.3%	5.35 [0.25, 116.31]
Xinyu Chen 2015 a	61	63	53	63	11.9%	5.75 [1.21, 27.44]
Yanqiong Jiang 2024	26	27	21	27	0.0%	7.43 [0.83, 66.62]
Zhi Li 2021	100	100	87	100	3.1%	31.01 [1.82, 529.29]
**Total (95% CI)**		**499**		**499**	**100.0%**	**5.32 [3.09, 9.14]**
Total events	483		422			

Heterogeneity: Chi² = 3.83, df = 8 (P = 0.87); I² = 0%
Test for overall effect: Z = 6.04 (P < 0.00001)

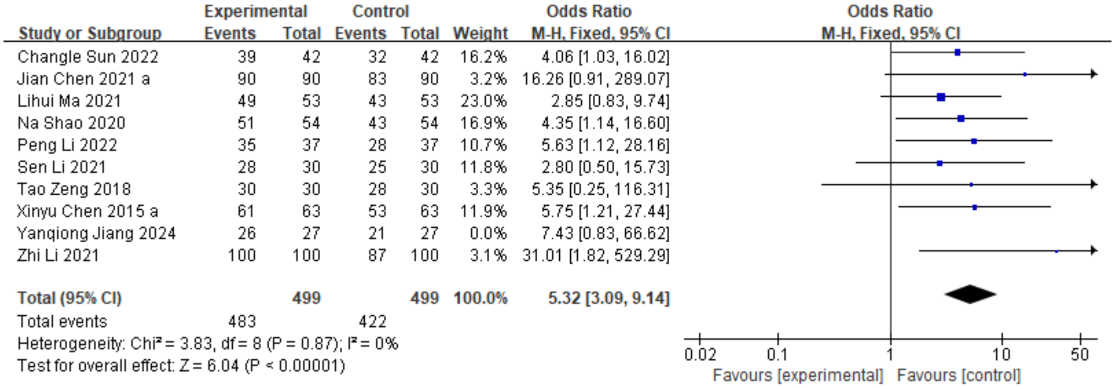

(j) Excluding Study J
