# Supplementary figures and images for: Effect of Medical Chitosan on Clinical Efficacy and Pain in Knee Osteoarthritis: A Systematic Review and Meta-Analysis
Source: Diseases. 2026 Jul 14;14(7):252. doi: 10.3390/diseases14070252 (PMC13408866; doi:10.3390/diseases14070252)

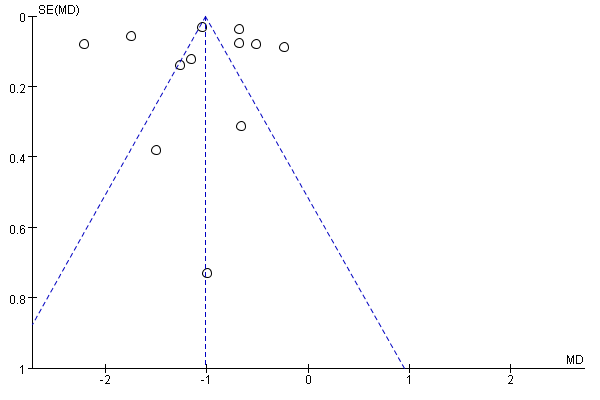

Supplement: Supplementary file 1 [file diseases-14-00252-s001.zip › Supplementary Figure S3. Funnel plot for the meta-analysis of VAS scores.png]

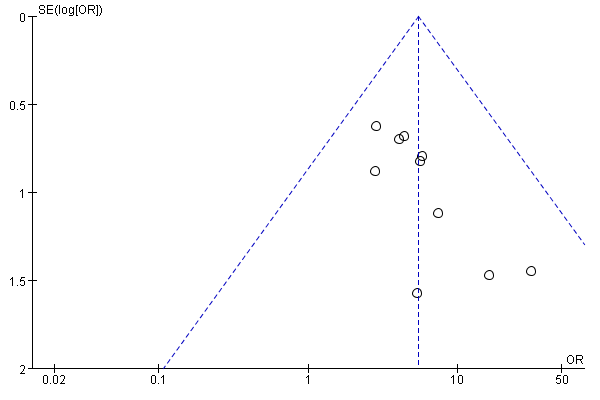

Supplement: Supplementary file 1 [file diseases-14-00252-s001.zip › Supplementary Figure S4. Funnel plot for the meta-analysis of clinical efficacy.png]
